# Culturomics of Bacteria from Radon-Saturated Water of the World’s Oldest Radium Mine

**DOI:** 10.1128/spectrum.01995-22

**Published:** 2022-08-24

**Authors:** Gabriela Kapinusova, Kunal Jani, Tereza Smrhova, Petr Pajer, Irena Jarosova, Jachym Suman, Michal Strejcek, Ondrej Uhlik

**Affiliations:** a University of Chemistry and Technology, Prague, Faculty of Food and Biochemical Technology, Department of Biochemistry and Microbiology, Prague, Czech Republic; b Military Health Institute, Ministry of Defence of the Czech Republic, Prague, Czech Republic; c University of Chemistry and Technology, Prague, Faculty of Food and Biochemical Technology, Department of Biotechnology, Prague, Czech Republic; State Key Laboratory of Microbial Resources, Institute of Microbiology, Chinese Academy of Sciences

**Keywords:** radioactive water springs, subsurface microbiology, environmentally relevant cultivation approaches, oxidative stress response, novel taxa, extremophiles

## Abstract

Balneotherapeutic water springs, such as those with thermal, saline, sulfur, or any other characteristics, have recently been the subject of phylogenetic studies with a closer focus on the description and/or isolation of phylogenetically novel or biotechnologically interesting microorganisms. Generally, however, most such microorganisms are rarely obtained in pure culture or are even, for now, unculturable under laboratory conditions. In this culture-dependent study of radioactive water springs of Jáchymov (Joachimstahl), Czech Republic, we investigated a combination of classical cultivation approaches with those imitating sampling source conditions. Using these environmentally relevant cultivation approaches, over 1,000 pure cultures were successfully isolated from 4 radioactive springs. Subsequent dereplication yielded 121 unique taxonomic units spanning 44 genera and 9 taxonomic classes, ~10% of which were identified as hitherto undescribed taxa. Genomes of the latter were sequenced and analyzed, with a special focus on endogenous defense systems to withstand oxidative stress and aid in radiotolerance. Due to their origin from radioactive waters, we determined the resistance of the isolates to oxidative stress. Most of the isolates were more resistant to menadione than the model strain Deinococcus radiodurans DSM 20539^T^. Moreover, isolates of the *Deinococcacecae*, *Micrococcaceae*, *Bacillaceae*, *Moraxellaceae*, and *Pseudomonadaceae* families even exhibited higher resistance in the presence of hydrogen peroxide. In summary, our culturomic analysis shows that subsurface water springs contain diverse bacterial populations, including as-yet-undescribed taxa and strains with promising biotechnological potential. Furthermore, this study suggests that environmentally relevant cultivation techniques increase the efficiency of cultivation, thus enhancing the chance of isolating hitherto uncultured microorganisms.

**IMPORTANCE** The mine Svornost in Jáchymov (Joachimstahl), Czech Republic is a former silver-uranium mine and the world’s first and for a long time only radium mine, nowadays the deepest mine devoted to the extraction of water which is saturated with radon and has therapeutic benefits given its chemical properties. This healing water, which is approximately 13 thousand years old, is used under medical supervision for the treatment of patients with neurological and rheumatic disorders. Our culturomic approach using low concentrations of growth substrates or the environmental matrix itself (i.e., water filtrate) in culturing media combined with prolonged cultivation time resulted in the isolation of a broad spectrum of microorganisms from 4 radioactive springs of Jáchymov which are phylogenetically novel and/or bear various adaptive or coping mechanisms to thrive under selective pressure and can thus provide a wide spectrum of capabilities potentially exploitable in diverse scientific, biotechnological, or medical disciplines.

## INTRODUCTION

Geothermally heated groundwater, such as thermal springs with temperatures higher than 37°C or less warm thermal springs reaching the surface with a temperature of at least 20°C, are natural phenomena that occur worldwide (1, 2). Since the final character of water springs can be influenced not just by the chemical composition, pH, temperature, and salinity (3, 4), but also by tectonic settings and subsurface bedrock (1), every single water spring can be considered to be a unique habitat. Even though microorganisms are able to exist wherever their minimal nutritional requirements are met (5), any changed variable (minerals, ions, carbon sources, etc.) in the surrounding conditions can lead to diversification of the microbial community (4, 6). Moreover, results from individual studies focusing on a genetic comparison of seemingly identical organisms, such as *Sulfolobus* strains with nearly identical 16S rRNA but differing in nine protein-coding loci (7), or different lineages of *Synechococcus* across geographical isolated environments (8), suggest that over time originally genetically identical microorganisms which exist and evolve in geologically distinct sites differentiate into new, no longer common species (5). Further evidence of slowly manifesting evolutionary nuances was provided by Klassen and Foght, 2011 (9) who characterized hymenobacters isolated from Victoria Upper Glacier which differed in their conserved and repetitive DNA segments and phenotype characteristics from already described, genetically nearly identical hymenobacters. Their divergence can be explained by long-term dormancy and the fact that ancient genotypes, conserved in the ice matrix, revive thanks to suitable conditions of the biosphere, and therefore coexist with modern genotypes at the same time (9). Thus, the hypothesis arises that 'ancient microorganisms' separated for a certain epoch (e.g., deep underground, in glaciers, etc.) can develop in a completely different way than microorganisms that constantly face surface conditions and, consequently, can start the evolution of a new phylogenetic branch.

The above-mentioned unique environmental sites with the possible presence of 'ancient microorganisms' are often extreme habitats. Therefore, these sites are attracting considerable attention in the context of the search for undescribed (9–11), thermoresistant (12, 13), acidophilic (14, 15), or otherwise interesting microorganisms. Due to unusual metabolic pathways, microorganisms isolated from extreme habitats are often used as biocatalysts or as a source of extremozymes and extremolytes (16). However, the isolation of such microorganisms is significantly complicated, for example, by the dormant state in which most of these microorganisms occur (17). Endeavoring to increase cultivation yields, several cultivation improvements have been implemented in methodical procedures, ranging from the 'provision of basic and specific needs' such as the addition of essential substances (nutrients, trace elements), growth factors such as Rpf (17, 18), cAMP, and homoserine lactones (19); through the usage of high-tech isolating devices such as micromanipulation equipment (20, 21), streaking pens (22) or ichips (23); to the application of knowledge gathered from metagenomic (24) and metatranscriptomic (25) analyses. It is not only sophisticated cultivation approaches that have borne fruit, but also simple cultivation strategies such as the application of oligotrophic media, possibly based directly on the environmental matrix and/or in combination with a culture temperature relevant to the environment from which the microbe is isolated (26–28).

A promising location both in terms of potentially taxonomically and/or biotechnologically novel microorganisms is the mine Svornost in Jáchymov (Joachimstahl), Czech Republic, a former silver-uranium mine and the world’s first and for a long time only radium mine, nowadays the deepest mine devoted to the extraction of water which is saturated with radon (^222^Rn ~ 24 kBq/l) (29) due to radium decay in Jáchymov subsoil, and has therapeutic benefits given its chemical properties. This healing water, which is approximately 13 thousand years old, is used under medical supervision for the treatment of patients with neurological and rheumatic disorders. The first radioactive water spring to be used was named Curie, followed by the springs called Agricola, C1, and Academician Běhounek. This geologically ancient site with very rare characteristics of its waters can be described as an inhospitable place for any form of life. Despite this, based on the extensive microbial analyses of similar sites (4, 30, 31), we predicted the presence of diverse microbial communities. Furthermore, due to their radioactive character, the waters were expected to host radiotolerant or radioresistant microorganisms.

With the above in mind, we applied a series of environmentally relevant cultivation methods leading to the isolation of a diverse spectrum of prokaryotic microorganisms from Jáchymov's radioactive water springs. We based our work on the presumption that microorganisms living within the springs can utilize low-carbon substrates typically found in oligotrophic environments. Moreover, in order to imitate the conditions of water springs, we used sterilized spring water as the liquid part of all media, or even as the medium itself. Using these modifications, we expected to isolate phylogenetically novel microorganisms. The cultivation yields of each method used were evaluated. Since the microorganisms living in Jáchymov's radioactive springs are constantly exposed to ionizing α-radiation which subsequently causes the intracellular generation of reactive oxygen species (ROS) (32, 33), the oxidative stress tolerance/resistance of isolates obtained from these springs can be assumed. Based on this presumption, we built up a functional analysis of isolated cultures where chemical compounds menadione (34–36) and hydrogen peroxide (34, 36, 37) were used as intracellular ROS inducers. The oxidative stress response of all isolates is provided herein.

## RESULTS

### Evaluation of cultivation media yields.

**FIG 1:**
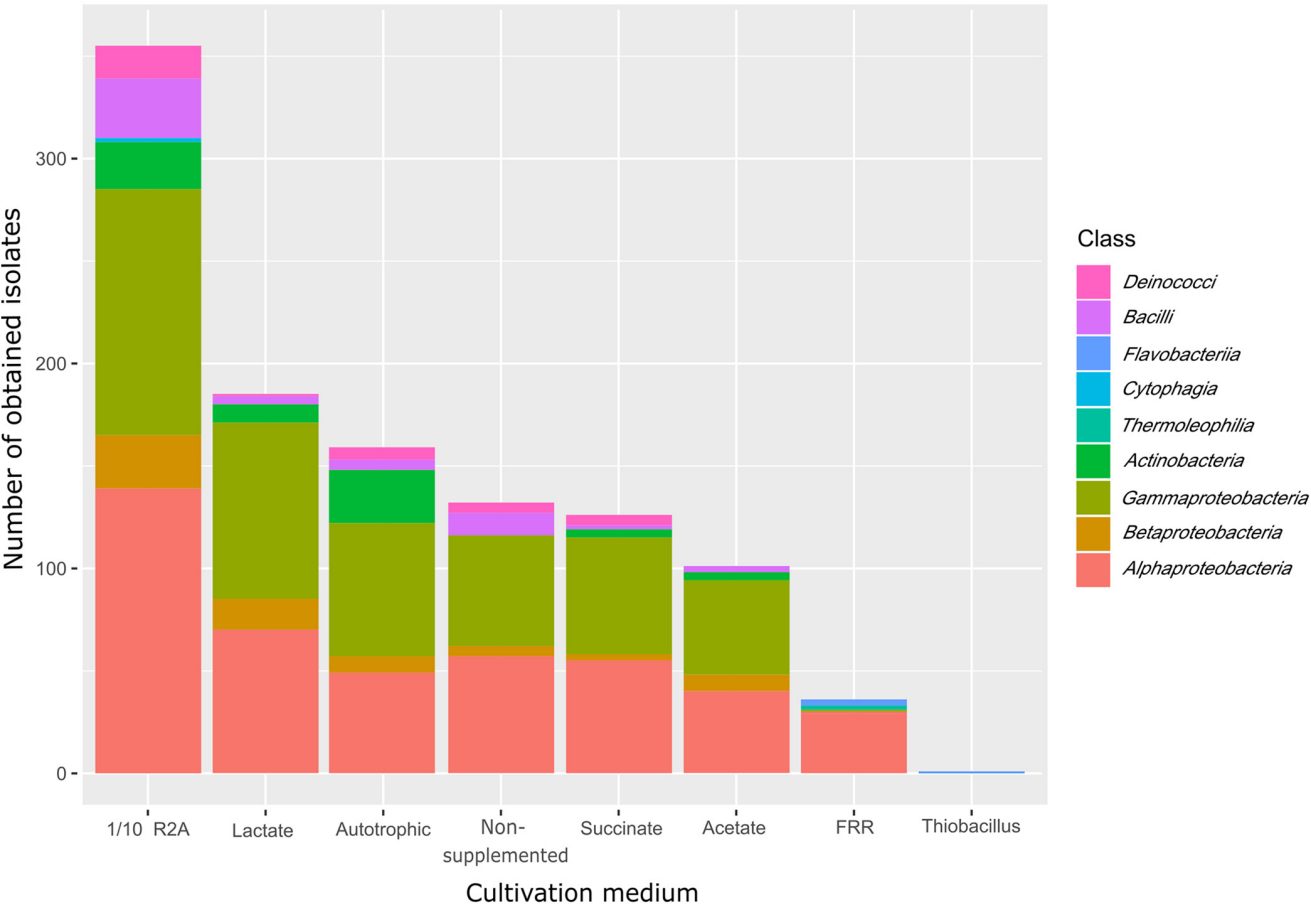
Determination of media cultivation yields and proportion of isolated bacteria at the class level for each medium used, compiled from 1,095 non-dereplicated isolates obtained here.

The media adjustments used here, such as the dilution of commercial media, use of water filtrate from the original source in media, low concentration of heterotrophic substrates, etc. (Table 1), resulted in the isolation of 1,095 pure cultures spanning 9 phylogenetic classes of 5 phyla (Fig. 1). The majority of the isolates were obtained from the Curie spring, followed by Běhounek, C1, and lastly, Agricola. Tenfold diluted R2A medium gave the greatest yield of 355 isolates (32% of the total number of isolates), while the nutrient-poor media had lower yields; lactate medium yielded 185 isolates (17%), non-supplemented medium 132 isolates (12%), succinate medium 126 isolates (12%), acetate medium 101 isolates (9%), and the Autotrophic medium yielded 159 isolates (15%). FRR-6, FRR-8 medium (hereinafter referred to as FRR) and Thiobacillus agar provided the lowest number of isolates, 36 isolates (3%) and only a single isolate, respectively. Importantly, phylogenetically novel isolates were obtained from 1/10 R2A (6 isolates), lactate medium (4), FRR medium (3), and one isolate for Thiobacillus agar and non-supplemented medium (Table 2).

**TABLE 1:** The media list with the chemical composition^*a*^

Medium	Composition	Reference
Acetate	Sodium acetate 0.5 g; Noble agar 18.0 g; water filtrate^*a*^ 1,000 mL	This study
Lactate	Aluminum L-lactate 0.5 g; Noble agar 18.0 g; water filtrate^*a*^ 1,000 mL	This study
Succinate	Disodium succinate 0.5 g; Noble agar 18.0 g; water filtrate^*a*^ 1,000 mL	This study
Non-supplemented	Noble agar 18.0 g; water filtrate^*a*^ 1,000 mL	This study
Thiobacillus	(NH4)_2_SO_4_ 0.10 g; K_2_HPO_4_ 4.00 g; KH_2_PO_4_ 4.00 g; MgSO_4_ × 7 H_2_O 0.10 g; CaCl_2_ 0.10 g; FeCl_3_ × 6 H_2_O 0.02 g; MnSO_4_ × H_2_O 0.02 g; Noble agar 18.00 g; Na_2_S_2_O_3_ × 5 H_2_O 10.00 g; water filtrate^*a*^ 1,000 mL	DSMZ no. 36, Germany
Autotrophic	(NH4)_2_SO_4_ 0.6 g; KH_2_PO_4_ 0.2 g; CaCl_2_.2H_2_O 0.04 g; MgS0_4_.7H_2_O 0.04 g; Ferric citrate 0.5 mg; phenol red 0.5 mg; Noble agar 18.0 g; distilled water 1,000 mL	Soriano and Walker, 1968 (113)
1/10 R2A	Casein acid hydrolysate 0.05 g; yeast extract 0.05 g; proteose peptone 0.05 g; starch soluble 0.05 g; glucose 0.05 g; dipotassium phosphate 0.03 g; magnesium sulfate 0.0024 g; Na-pyruvate 0.03 g; Noble agar 18.0 g; water filtrate^*a*^ 1,000 mL	Merck, United States
FRR	Yeast extract 1.0 g; proteose peptone 1.0 g; casamino acids 1.0 g; glucose 1.0 g; NaCl 1.0 g; Na-pyruvate 0.5 g; MgSO_4_ × 7 H_2_O 0.1 g; K_2_HPO_4_ 0.6 g; Noble agar 18.0 g; water filtrate^*a*^ 1,000 mL	DSMZ no. 822, Germany

a Four variants of each medium were prepared, i.e., separately for each water filtrate of Agricola, Běhounek, C1, and Curie spring.

**TABLE 2:** List of phylogenetically novel isolates

Isolate	Taxonomic classification	The closest type strain based on the 16S rRNA gene sequence similarity	Similarity (%)^*a*^	Origin^*b*^	Medium^*c*^	Novelty level	Accession no.^*d*^
J193	*Hymenobacter* sp.	*Hymenobacter russus* BT18^T^	98.07	Agricola	Thiobacillus agar	Novel species	JAJONE000000000
J219	*Rhizobacter* sp.	Rhizobacter profundi DS48-6-5^T^	97.82	Běhounek	1/10 R2A lactate	Novel species	JAJOND000000000
J223	*Aquabacterium* sp.	*Aquabacterium pictum* W35^T^	97.32	Běhounek	1/10 R2A	Novel species	CP088297
J276	*Aquincola* sp.	*Aquincola rivuli* KYPY4^T^	98.6	Běhounek	lactate	Novel species	JAJONC000000000
J315	*Sphingomonas* sp.	*Sphingomonas suaedae* XS-10^T^	98.93	C1	1/10 R2A lactate Non-supplemented	Novel species	CP088296
J344	*Sphingomonas* sp.	*Sphingomonas suaedae* XS-10^T^	99.29	C1	1/10 R2A	Novel species	JAJONB000000000
J367	*Phenylobacterium* sp.	Phenylobacterium immobile *strain* E^T^	98.25	C1 Curie	FRR	Novel species	JAJONA000000000
J372	*Flavobacterium* sp.	*Flavobacterium album* HYN0059^T^	97.37	C1	FRR	Novel species	JAJOMZ000000000
J379	Unclassified *Parviterribacteraceae* Bacterium	Parviterribacter kavangonensis D16/0/H6^T^	95.96	C1	FRR	Novel genus	CP088295
J426	*Phenylobacterium* sp.	Phenylobacterium muchangponense A8^T^	98.38	Curie	1/10 R2A	Novel species	JAJOMY000000000
J428	*Mesorhizobium* sp.	Mesorhizobium qingshengii CCBAU 33460^T^	98.30	Curie	1/10 R2A	Novel species	JAJOMX000000000
J452	Pseudomonas sp.	Pseudomonas *campi* S1-A32-2^T^	99.86	Curie	lactate	Novel species	CP088294

a Similarity (%) indicates the percentual 16S rRNA gene sequence match with the closest type strain.

b Origin describes the water spring of origin.

c Medium indicates the list of media with which the strains were cultured.

d All genomes are available in the NCBI assembly database under the indicated accession numbers.

### Phylogenetic diversity of bacterial isolates.

**FIG 2:**
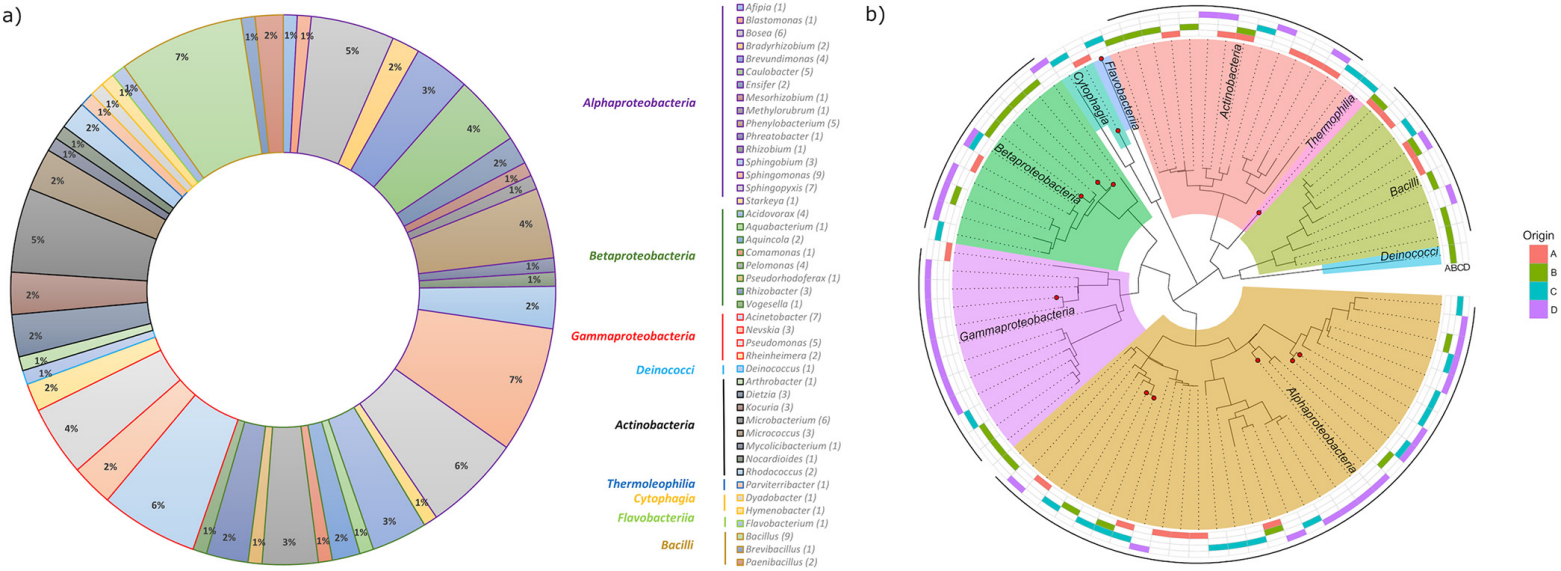
(a) Relative abundance of dereplicated isolates (at the level of genus and class) in 4 radioactive water springs. (b) Phylogenetic tree showing evolutionary relationship of isolates. This Maximum Likelihood phylogenetic tree was constructed based on trimmed partial 16S rRNA gene sequences (V2-V4 regions, ~590 bp). Phylogenetically novel isolates are highlighted with red dots. The outer circle depicts the origin of isolates. A = Agricola, B = Běhounek, C = C1, D = Curie.

After the dereplication using MALDI-TOF MS and 16S rRNA gene sequencing, 121 unique isolates were identified which spanned 44 genera from the classes *Alphaproteobacteria*, *Betaproteobacteria*, *Gammaproteobacteria*, *Cytophagia*, *Flavobacteriia*, *Actinobacteria*, *Thermoleophilia*, *Bacilli* and *Deinococci* (Fig. 2a and Table S2). The highest number of isolates (6 to 9) was obtained within the genera *Bacillus*, *Bosea*, *Sphingomonas*, *Sphingopyxis*, Acinetobacter, and *Microbacterium* (Fig. 2a), and the rest of the genera were mostly represented by only 1 or 2 members. None of the isolates were isolated simultaneously from all 4 water springs, and only members of the genus *Bacillus* were isolated from all 4 springs. Moreover, the majority of species were only isolated from a single spring, making co-isolation across multiple springs relatively exceptional. The majority of unique isolates were obtained from the Curie spring, followed by Běhounek (Fig. 2b). Twelve out of 121 dereplicated bacterial isolates (Table 2) exhibited lower 16S rRNA gene sequence similarity to the closest type strain cataloged in the RefSeq RNA database (NCBI) NCBI database and EZBioCloud 16S rRNA gene sequence database than the cut-off for delineating a separate species, and were taxonomically classified in more detail upon whole-genome sequencing.

### Whole-genome sequencing of phylogenetically novel isolates.

Whole genomes of 12 bacterial strains potentially representing novel taxa (the 16S rRNA gene similarity with the closest type strain ≤98.5%) were sequenced using the ONT MinION platform. At the level of phyla, these 12 strains were classified into *Proteobacteria* (*n* = 9), *Bacteroidetes* (*n* = 2), and *Actinobacteria* (*n* = 1). The only isolate which was novel at the genus level was isolated from radioactive water spring C1 (radioactivity 11 kBq/l) on the FRR medium, and was designated J379. The novelty of this isolate at the genus level is apparent from (i) the significantly low 16S rRNA sequence similarity of 95.19% to its closest taxonomic neighbor Parviterribacter kavangonensis D16/0/H6^T^, which belongs to class *Thermoleophilia* of phylum *Actinobacteria*; (ii) distinct clustering of isolate J379 from the members of the family *Parviterribacteraceae* (Fig. S3), as shown by the core gene-based phylogenetic analysis; and (iii) low POCP (≤48.1%) and ANI (≤77.9%) values. With that in mind, isolate J379 is presented here as a novel species of a novel genus of the family *Parviterribacteraceae*.

The 16S rRNA gene similarity of the remaining 11 isolates to the closest type strains was lower than the suggested species delineation cut-off 98.5%. The core gene-based phylogenetic assessment revealed that all 11 isolates formed distinct branches from their related taxa (Fig. S2 to S11). The core gene phylogeny of the 2 isolates J315 and J344 affiliated to *Sphingomonas*, sharing ANI similarity of 99.9%, were collectively processed and depicted in Fig. S5. Similarly, the POCP and ANI values of these isolates and their related members were substantially lower than the suggested threshold of taxa divergence (Table S3). It is noteworthy that the DNA G+C content of only 1 novel isolate was below 50% (*Flavobacterium* sp. J372, 42.4%). In fact, the G+C content was >70% in 3 isolates, with a maximum value of 71.7% in isolates J379 and J223, and >65% in 6 isolates.

### Oxidative stress response determination.

**FIG 3:**
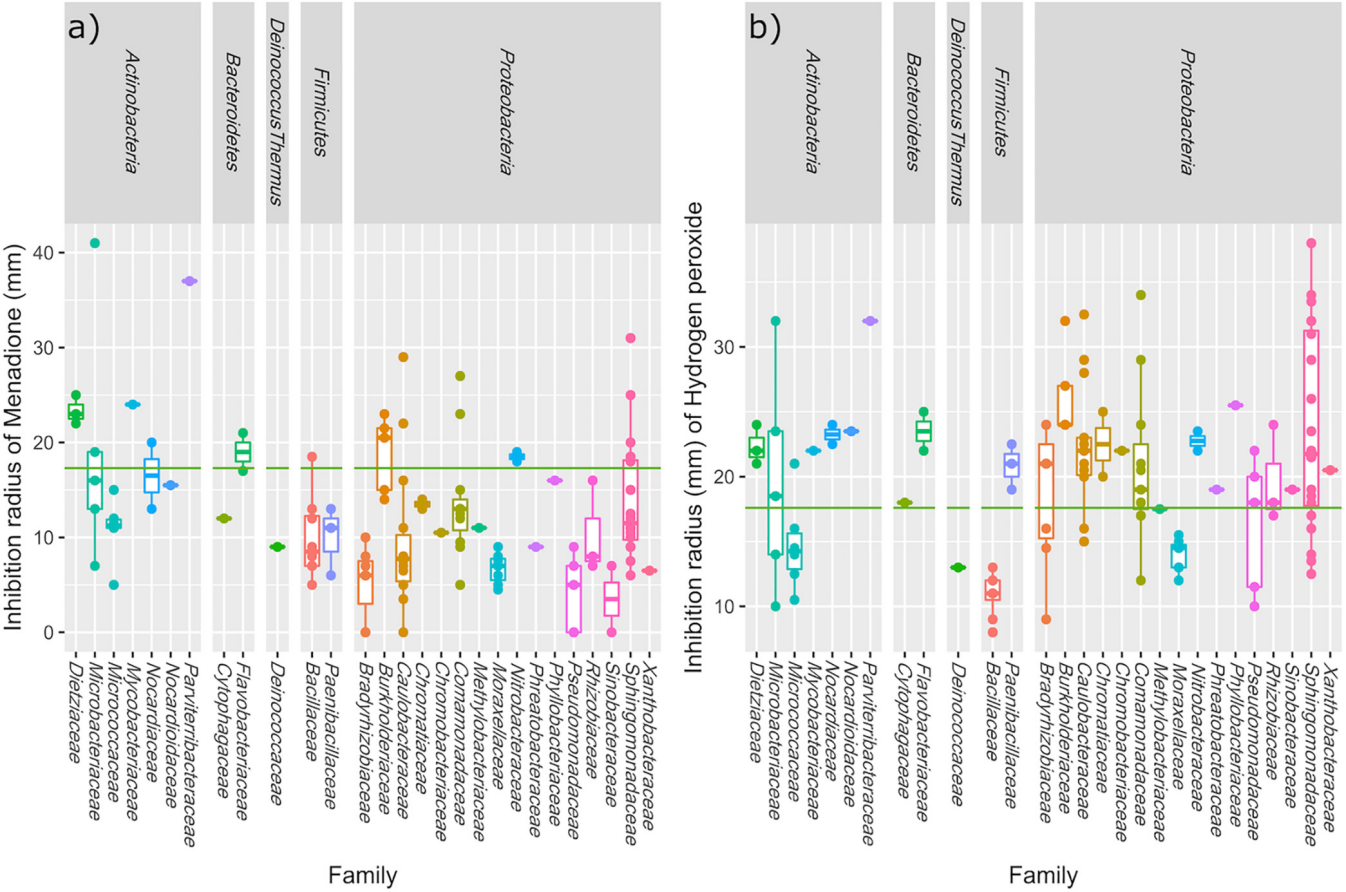
Response of bacterial isolates to menadione (a) and hydrogen peroxide (b). The response of individual isolates belonging to a particular family are plotted, with the ability of isolates to resist the oxidative stress deduced as an inhibition zone radius (mm) created around the soaked sterile discs. The boxplots were constructed from the values grouped according to their family members. The green line indicates the radius of the inhibition zone for the reference strain Deinococcus radiodurans DSM 20539^T^.

In order to confirm the hypothesis that microorganisms living in the radioactive water springs of the Svornost mine are able to sustain oxidative stress, we conducted a functional assay of the oxidative stress response of the isolated cultures to menadione (80 μM) and hydrogen peroxide (9.8 M). The growth inhibition zones of the reference strain D. radiodurans DSM 20539^T^ were very similar for menadione and hydrogen peroxide, 18 and 17 mm, respectively. The majority of the isolates were more resistant to menadione than D. radiodurans DSM 20539^T^, with the most resistant isolates having an inhibition zone radius of 0–5 mm. These isolates belonged to the *Pseudomonadaceae*, *Sinobacteraceae*, *Bradyrhizobiaceae*, and *Caulobacteraceae* families. In addition, hydrogen peroxide appeared to be a stronger growth inhibitor than menadione (Fig. 3), with the most resistant isolates being those of the genera Bacillus, Bosea, Microbacterium, and Pseudomonas. Several isolates from the families *Micrococcaceae*, *Bacillaceae*, *Moraxellaceae*, *Pseudomonadaceae* and even the isolate of the *Deinococcus* genus isolated from Běhounek spring exhibited higher resistance to both stress agents than D. radiodurans DSM 20539^T^.

### Features of the endogenous defense system in the phylogenetically novel taxa.

**FIG 4:**
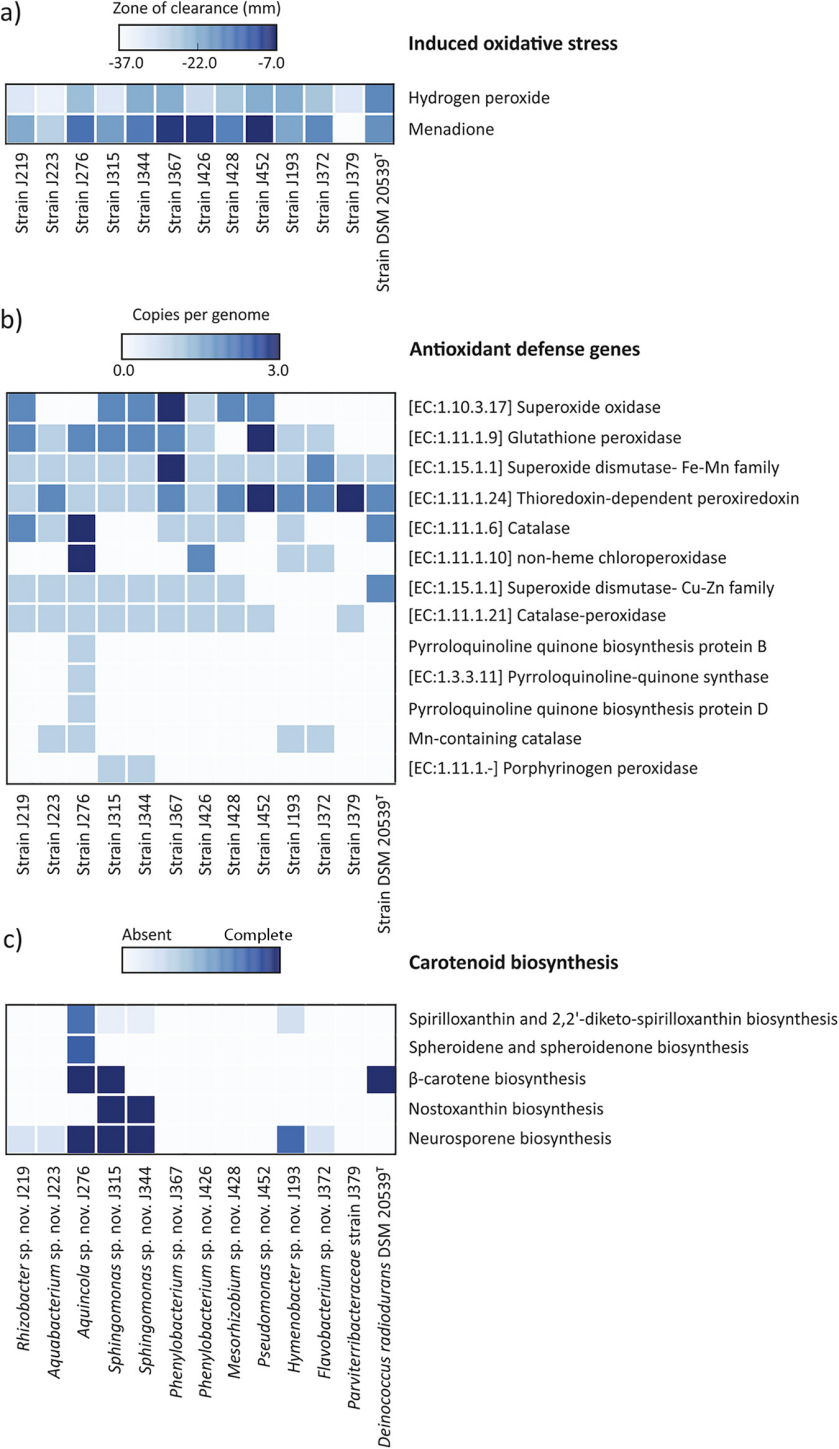
Response to induced oxidative stress as zone of clearance upon exposure of strains to hydrogen peroxide and menadione (a) and presence of genes (shown as a gene copy per genome) involved in endogenous defense mechanism (EDM), including antioxidant defense genes (b) and completeness of diverse carotenoid biosynthesis pathway (c) in genomes of phylogenetically novel taxa from Jáchymov’s radon water springs.

Seven out of the 12 phylogenetically novel isolates were more resistant to menadione than D. radiodurans DSM 20539^T^, with those affiliated to the genera Pseudomonas (J452) and Phenylobacterium (J367 and J426) exhibiting the highest resistance to menadione (Fig. 4a). Fe-Mn superoxide dismutase and thioredoxin-dependent peroxiredoxin, which are part of the antioxidant defense genes that enable the elimination of O_2_- and organic hydroperoxides (OHPs) were found to be present in all phylogenetically novel isolates (Fig. 4b). Phenylobacterium sp. J367 contained 3 copies of the Fe-Mn superoxide dismutase gene, whereas Pseudomonas sp. J452 and Parviterribacter sp. J379 contained 3 copies of thioredoxin-dependent peroxiredoxin in their genomes. The genome of Aquincola sp. J276 contained a diverse group of antioxidant defense genes when compared to other isolates. Assessment of the glutathione-mediated quenching of ROS revealed γ-glutamyltranspeptidase and isocitratedehydrogenase [NADP] genes in all the strains except for Sphingomonas sp. J344 and Phenylobacterium sp. J367, respectively (Fig. S12c). The glutathione peroxidase gene was present in 3 copies in the Pseudomonas sp. J452 genome, but was absent in 2 of the isolates (J379 and J428), as well as in the reference strain D. radiodurans DSM 20539^T^. In summary, the bacterial strains Pseudomonas sp. J452, Phenylobacterium sp. J367, Phenylobacterium sp. J426, and Aquincola sp. J276 with abundant stress response genes also had the highest tolerance to hydrogen peroxide and menadione (Fig. 4 a and b, and Fig. S12 a and b).

Diverse carotenoid biosynthesis pathways were detected in the genome of isolate J276 (Fig. 4c). Neurosporene biosynthesis pathway was found in isolates J193, J276, J315, and J344, whereas β-carotene biosynthesis was detected in isolates J276 and J315. Furthermore, genes encoding the biosynthetic pathways of spheroidene and spheroidenone as well as spirilloxanthin and 2,2’-diketo-spirilloxanthin were detected in the genome of isolate J276. Nostoxanthin biosynthesis was found in the genomes of isolates J315 and J344 (Fig. 4c). DNA repair genes were ubiquitously present in all the isolates, including those encoding recombination protein RecA, single-stranded-DNA-specific exonuclease RecJ, DNA helicase UvrD/PcrA, and DNA recombination and repair proteins RecO and RecR (Fig. S12c). The key DNA repair and recombination protein RadA was present in 8 isolates (Fig. S12c). It is noteworthy that the gene for multifunctional exodeoxyribonuclease III was present in all isolates, but absent in Flavobacterium sp. J372. Similarly, genes encoding for DNA mismatch repair proteins MutL and MutS were found in all the isolates except for Parviterribacteraceae strain J379.

**FIG 5:**
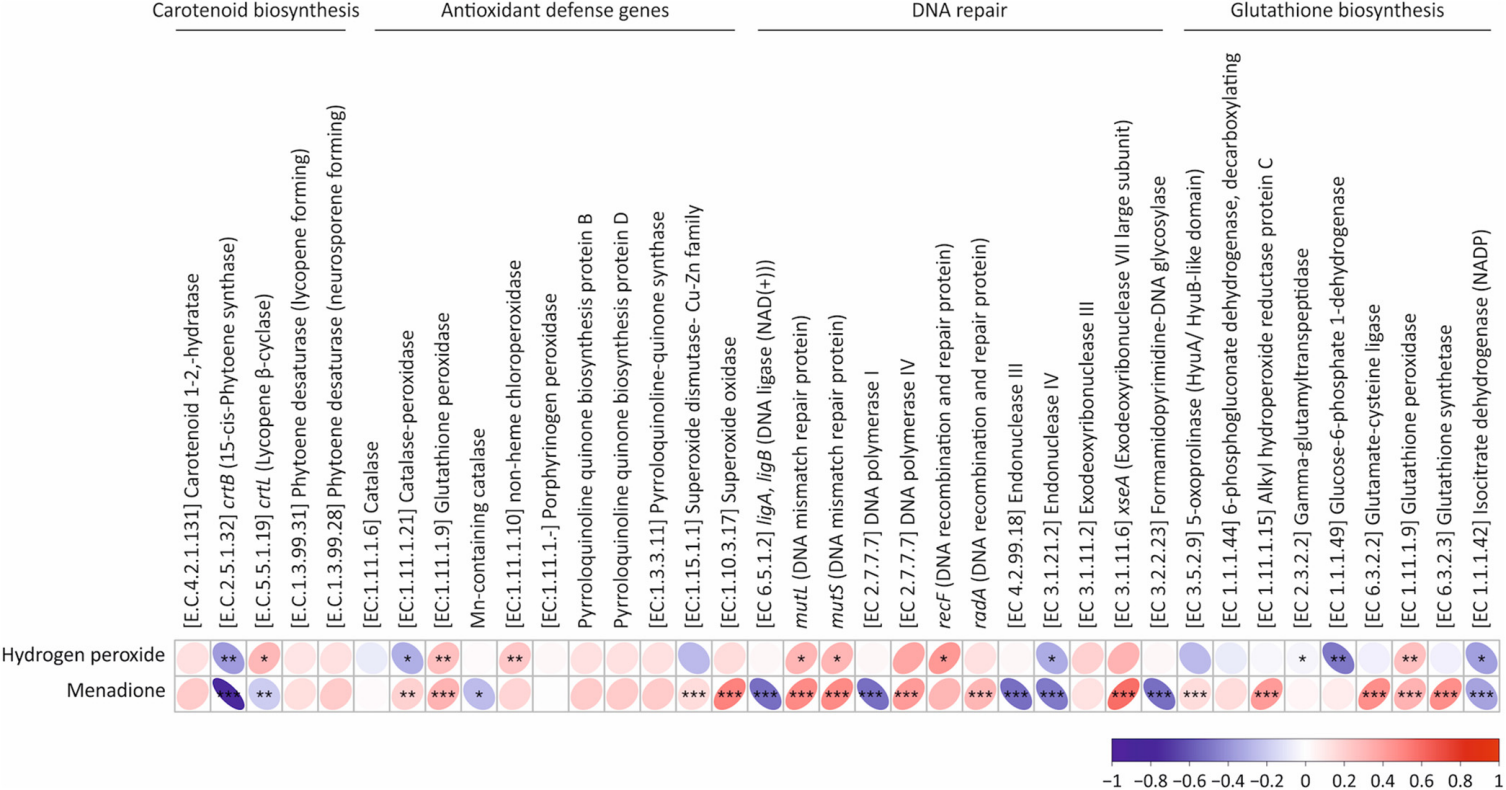
Spearman's rank correlation analysis representing relationship between response to induced oxidative stress (menadione and hydrogen peroxide) and the presence of endogenous defense genes. The positive correlation (RED) implies a proportionate abundance of the genes and thereby an active response toward the induced oxidative stress and vice-versa. Significant correlations are denoted with an asterisk (where ***, *P* ≤ 0.001; **, *P* ≤ 0.01; *, *P* ≤ 0.05).

The Spearman's correlation revealed a potential role of endogenous defense mechanism (EDM) genes in response to oxidative stress (Fig. 5). The presence of genes that were positively correlated with tolerance to both hydrogen peroxide and menadione included: *mutL*, *mutS* (DNA mismatch repair protein) (*P ≤ *0.05 and *P ≤ *0.001, respectively), and glutathione peroxidase (*P ≤ *0.01 and *P ≤ *0.001, respectively). A negative correlation was observed for endonuclease IV (*P ≤ *0.05 and *P ≤ *0.001, respectively) and isocitrate dehydrogenase (NADP) (*P ≤ *0.05 and *P ≤ *0.001, respectively). The presence of the *recF* gene (DNA recombination and repair protein) (*P ≤ *0.05) was positively correlated with tolerance to hydrogen peroxide, whereas catalase-peroxidase (*P ≤ *0.05), γ-glutamyltranspeptidase (*P ≤ *0.05) and glucose-6-phosphate 1-dehydrogenase (*P ≤ *0.01) were correlated negatively. Similarly, the genes for catalase-peroxidase (*P ≤ *0.05), the superoxide dismutase-Cu-Zn family (*P ≤ *0.001), DNA polymerase IV (*P* ≤ 0.001), *radA* (DNA recombination and repair protein) (*P ≤ *0.001), 5-oxoprolinase (*P ≤ *0.001), glutamate-cysteine ligase (*P ≤ *0.001), and glutathione synthetase (*P ≤ *0.001) were positively correlated with tolerance to menadione, whereas *crtL* (Lycopene β-cyclase) (*P ≤ *0.01), Mn-containing catalase (*P ≤ *0.05), DNA polymerase I (*P ≤ *0.001), endonuclease III (*P ≤ *0.001), and formamidopyrimidine-DNA glycosylase (*P ≤ *0.001) were correlated negatively.

## DISCUSSION

Radionuclides in subsurface waters are a common phenomenon on Earth. The majority of radioactive elements in water come from uranium, thorium, and actinium series, with the most abundant elements being ^238^U, ^236^Ra, and ^232^Th (38). Radon, produced by the radioactive decay of uranium and radium, is a very frequent radioactive gas contained in water springs (39–41), an example being Jáchymov's water springs. In general, springs with very low (tens of Bq/l) or even negligible (tenths of Bq/l) concentrations of ^222^Rn are frequently found in nature (41–43), however higher concentrations of ^222^Rn ranging from hundreds of becquerel (44, 45) to units of kilobecquerel per L (46) are less common and concentrations of ^222^Rn in tens of kilobecquerels per L are rarely found in the world. Among these exceptional sites are the Stripa granite groundwaters (Sweden), with an ^222^Rn concentration of ~100 kBq/l (40, 47), and also the highly radioactive water springs of Jáchymov studied here, with the concentration of ^222^Rn reaching up to 24 kBq/l (29).

In general, due to their deep location and inaccessible terrain, underground water springs are rarely explored, and so their microbial colonization remains hidden. So far, only a small number of radioactive water springs have been subjected to microbial screening (48–50), and extremely little is known about the composition of the microbial communities of underground radioactive springs. Nevertheless, the aforementioned microbial analyses of thermal (51) and underground non-radioactive springs (52) confirmed the presence of diverse rock-dwelling bacteria belonging to various clades, many of which had been unexplored. The study of Enyedi, 2019 (50) in Hungarian radioactive springs even revealed the presence of previously unidentified taxa of *Chloroflexi*, *Nitrospirae*, *Proteobacteria*, *Planctomycetes*. In addition, the application of a series of modified cultivation approaches led to the isolation of various novel bacterial species (50). The 16S rRNA gene-based and metagenomic analyses also proved the presence of several members of *Archaea* in many water springs (51–54). In fact, extreme aquatic biomes have previously appeared to be a promising source of novel prokaryotic taxa, such as Obsidian Pool in Yellowstone National Park (55) from which the novel 16S rRNA gene sequences of the bacterial phylum *Armatimonadetes* (56), previously termed OP10, were recovered. A member of the novel bacterial phylum *Caldiserica* (57) (former candidate phylum OP5) was also first described in a hot spring in Yellowstone National Park (55). In this report, a culturomic survey was conducted of four springs from the world’s oldest radium mine devoted to the extraction of radon-saturated water by applying ‘elemental’ culture procedures using media containing substrates relevant to oligotrophic environments (Fig. 1). These results clearly support the hypothesis that extreme environments, such as the radon waters analyzed here, host phylogenetically novel microorganisms (Fig. 2b and Table 2). Our findings correspond with previously performed culture-dependent studies of radioactive sites, where novel bacterial species were found (58, 59). Moreover, the assumption that radon water populations are able to resist oxidative stress was confirmed (Fig. 3); some 30% of the tested isolates, in particular those from the *Micrococcaceae*, *Deinococcaceae*, *Bacillaceae*, and *Moraxellaceae* families, exhibited lower sensitivity to hydrogen peroxide than D. radiodurans DSM 20539^T^, which was reported to be highly resistant to oxidative stress (60). In addition, the vast majority of the isolates were more resistant to menadione (80 mM) than D. radiodurans DSM 20539^T^ (Fig. 3). Even the isolate of the genus Deinococcus (strain J250) was more resistant to both hydrogen peroxide and menadione (Fig. 3).

Bringing environmental microorganisms to pure culture remains very difficult (17), due to their adaptation to low concentrations of nutrients or their possible occurrence in the dormant phase. Generally, the usage of conventional or nutrient-rich media mostly leads to the acquisition of a less diverse set of fast-growing bacteria rather than heterogeneous communities comprising both fastidious and slow-growing ones (27, 61, 62). With that in mind, in our study specific cultivation procedures such as prolonged cultivation time, highly diluted inocula, and relatively low concentrations of growth substrates were employed, enabling the isolation of pure cultures related to taxa reported only recently, such as Nevskia lacus (63), Aquincola rivuli (64), Phreathobacter stygius (65), or even 12 species that have not been isolated yet (Table 2). Although we mainly isolated members of *Proteobacteria* (α-, β-, γ-), followed by *Actinobacteria*, *Firmicutes*, *Bacteroidetes*, and *Deinococcus-Thermus* phyla, the diversity of the isolates was much greater than those reported from hot springs, possibly due to a more diverse set of culturing approaches. For instance, using only nutrient agar medium, Sen and Maiti, 2014 (66) retrieved less than 10 bacterial genera, predominantly composed of Bacillales, and Narsing Rao, 2021 (31) isolated members of 12 genera using 6 different media. Contrarily, Enyedi, 2019 (50) isolated 452 bacterial strains from biofilm samples of the Gellért Hill discharge area of the Buda Thermal Karst System (Hungary) and identified 51 genera (versus 44 in this study) and at least 8 potentially novel species (versus 12 in this study) while using environment-mimicking strategies. Some isolates in our study belonged to families usually found in soils or rhizosphere, such as *Bradyrhizobiaceae* and *Rhizobiaceae* (Fig. 2a), yet their isolation from subsurface waters is in accordance with the study of Pedron, 2019 (67), who isolated populations of these families from Italian thermal springs. In fact, members of *Bradyrhizobiaceae* and *Rhizobiaceae* as well as other bacteria living in aquifers can naturally occur in sediments or on rock surfaces attached to mineral particles (67), and thus their presence in spring water samples can be expected. Numerous studies reported an increase in the diversity of their isolates, including those hitherto uncultured, when cultivation strategies relied on mimicking the conditions experienced at a particular site (68, 69) or by performing the cultivation in that same environment (23). In line with the hypothesis that Jáchymov's very deep and ancient radon waters are extremely nutrient-poor matrices where microorganisms utilize inorganic substrates from the surrounding rock and water or utilize scarce organic matter that has infiltrated into the underground through groundwater, we used plain spring water as the main component of the cultivation media. Since we obtained more than 130 isolates using this non-supplemented groundwater-based medium (Fig. 1), we suppose that this imitation of the environment contributes to increased cultivation yields similarly to Wu, 2020 (70). Diluted R2A medium, traditionally used for the cultivation of environmental microbes (71–73), was also found to be effective for the cultivation of a broad range of Jáchymov's spring microbes (Fig. 1). In terms of the isolation of previously unidentified taxa, the spring-water-amended 1/10 R2A and lactate medium turned out to be the most suitable for such cultivation, yet members of novel species were also isolated on FRR and Thiobacillus agar (Table 2). Our results thus indicate that various types of media should be applied in searches for phylogenetically novel taxa from oligotrophic environments.

Members of 11 potentially novel species spanning the *Proteobacteria* and *Bacteroidetes* phyla, and a member of a putative novel genus belonging to the *Parviterribacteraceae* family (Table 2 and Fig. S1–S11) were isolated and characterized. The closest relatives of some of the newly described taxa were previously isolated from aquatic environments, such as Rhizobacter profundi DS48-6-5^T^ (74), isolated from freshwater sediment in Korea; Aquabacterium pictum W35^T^ (75), isolated from Tama River in Japan; Aquincola tetriaricarbonis L10^T^ (76), isolated from contaminated groundwater (Germany); or Flavobacterium album HYN0059^T^ (77), isolated from a freshwater lake (Korea). Other closest relatives were described in different types of soils, such as Hymenobacter psychrotolerans Tibet-IIU11^T^ (78) in permafrost sediment in China, Sphingomonas suaedae XS-10^T^ (79) in the rhizosphere of Suaeda salsa (China), Phenylobacterium immobile E^T^ (80) in chloridazon-contaminated soil, and Phenylobacterium muchangoponense A8^T^ (81) in beach soil (Korea), or Pseudomonas campi S1-A32-2^T^ (82) in grassland soil. Mesorhizobium qingshengii CCBAU 33460^T^ (83), the closest type strain to isolate J428, was isolated from root nodules of Astragalus sinicus. Our study thus contributes to a growing body of studies that report radioactive sites and water springs as sites with as-yet uncultured or unidentified microorganisms, often with desired capabilities, such as UV-radiation resistant Deinococcus sahariensis isolated from water springs in the Tunisian Sahara (84), or γ radiation-tolerant Sphingomonas jaspsi isolated from fresh water in the Misasa region in Japan, which is known for its high natural radioactivity (85).

Radioactive radiation causes serious genomic damages in exposed microorganisms by the direct interaction of γ-photons with DNA, and mainly indirectly by the generation of superoxide radicals, in the form of ROS, resulting in oxidative stress (86). Such ROS, predominantly O_2_-, OH-, and NO-, damage the structures of various biomacromolecules and thus disrupt their function (86). Radiation resistance in microorganisms is maintained by several mechanisms. Deinococcus radiodurans, which is reported to be the most radio-resistant bacterial species (60, 87), uses a highly efficient DNA repair system (88). Apart from that, a recent study by Yang, 2021 (86) suggested that D. radiodurans possesses 2 levels of bacterial EDM against radiation, namely structural adaptations, such as the structure of cell membranes, cell envelopes, genome, etc., and non-structural adaptations, such as cell components scavenging or quenching ROS (86). Among the key components of EDM, carotenoids have been proposed to be crucial in the reduction of radiation-induced membrane damage (86). Besides these, various classes of proteins involved in redox processes have been identified to play a role in EDM, including enzymes such as superoxide dismutases, catalases, peroxidases, glutathione peroxidases, peroxiredoxin, and coenzyme pyrroloquinoline-quinone (89–92). Additionally, the synthesis of glutathione in bacteria has been shown to influence endogenous enzymatic antioxidants for redox regulation, which in turn induce tolerance of oxidative stress (93, 94). Finally, increased exposure to radiation increases the burden of the mutagenesis, and its mitigation necessitates an effective DNA repair mechanism (95).

The high radon level in Jáchymov's water springs very likely represents conditions that have forced the indigenous microorganisms to undergo some of the above specific adaptations. Radio-resistant microorganisms are often co-resistant, or at least less sensitive, to other types of DNA- or protein damage caused by UV-irradiation, desiccation, exposure to toxic metals, high temperatures (50), or by certain chemical compounds such as menadione or hydrogen peroxide that were also used here as oxidative stress inducers. Many of our isolates proved to be more tolerant to menadione than the reference strain D. radiodurans DSM 20539^T^, and some were more resistant to hydrogen peroxide (Fig. 3). The greater resistance to oxidative stress of the isolates studied here might be also ascribed to several other causes. For example, the isolates of the genus Bacillus intrinsically contain multiple copies of the menaquinone MK-7 (96), which are involved in the respiratory electron transport chain, and their reduced forms protect the cellular membranes against oxidation (97). The diverse pigmentation of Brevundimonas (orange), Deinococcus (pink), Methylorubrum (purple), Kocuria (yellow), and Sphingopyxis (yellow-orange) strains, which is often the result of the presence of various carotenoids, can play a pivotal role in the response to radiation, as in other UV/α/β/γ radiation-resistant microorganisms such as red-pigmented Deinococcus radiodurans (87, 98) and Rubrobacter radiotolerans (99, 100), or orange-red-pigmented Brevundimonas sp. ZF12 (58). Carotenoids, being radical scavengers, have a direct protective role in quenching oxidative stress in cells. Their importance was confirmed by the study by Zhang, 2007 (101) and the follow-up study by Yang, 2021 (86), where mutants of the type strain D. radiodurans DSM 20539^T^ exhibited increased sensitivity to oxidative stress. In this regard, the genomic analysis of colored phylogenetically novel bacterial isolates, including pink-colored isolates J193 and J276 or yellow-colored J315 and J344, revealed diverse carotenoid biosynthesis pathways, with neurosporene biosynthesis being present in all four isolates (Fig. 4). The biosynthesis of β-carotene, spheroidene, spheroidenone, spirilloxanthin, and 2,2’-diketo-spirilloxanthin were other major carotenoid biosynthesis pathways detected in these isolates (Fig. 4).

The phylogenetically novel bacterial isolates were mostly rich in G+C content. In fact, 9 out of 12 isolates had a G+C content of ≥65% (Table S3). The higher environmental temperature has been associated with increased heat-induced mutagenesis, resulting in increased DNA repair activity and G+C content in microorganisms (95). Thus, the observed G+C content of the bacterial isolates is very likely part of an EDM to tolerate heat and/or radiation. The genomic analyses also showed that the isolates harbor a wide spectrum of genes encoding for ROS-scavenging enzymes (Fig. 4b). The potential of the isolates for withstanding oxidative stress was supported by the analysis based on Spearman’s rank correlation between the presence or absence of EDM genes and the response to oxidative stress (Fig. 5). One of the genes positively correlated with the response to hydrogen peroxide and menadione was glutathione peroxidase (*P ≤ *0.01 and *P ≤ *0.001, respectively) (Fig. 5). Similarly, antioxidant genes such as those encoding catalase-peroxidase (*P ≤ *0.05) and the superoxide dismutase-Cu-Zn family (*P ≤ *0.001) were also positively correlated with tolerance of menadione. The presence of diverse catalases, peroxidases, superoxide dismutases, and glutathione peroxidases has been extensively reported to reduce the deleterious impact of oxidative stress on macromolecules (102–107). Additionally, a positive correlation between hydrogen peroxide or menadione tolerance and the presence of genes encoding MutL, MutS (DNA mismatch repair protein) (*P ≤ *0.05 and *P ≤ *0.001, respectively) emphasize the role of DNA repair in the adaption to this stress (95). The DNA recombination and repair protein RadA (*P ≤ *0.001) was also positively correlated wiht the response to menadione. The DNA recombination and repair protein RadA, along with the recombination protein RecA, have been demonstrated to play a key role in extensive DNA repair (108). Although the recombination protein RecA was present in all the isolates, the absence of DNA recombination and repair protein RadA in four isolates might have a detrimental impact (Fig. S12). In fact, our isolates with missing *radA* genes were less tolerant to hydrogen peroxide (Fig. 4a and Fig. S12). Similarly, the absence of DNA mismatch repair proteins, namely, MutL and MutS in *Parviterribacteraceae* strain J379, could be coupled with the observed limited tolerance to oxidative stress. The positive correlation of the presence of genes responsible for glutathione synthesis, such as glutamate-cysteine ligase (*P* ≤ 0.001) and glutathione synthetase (*P ≤ *0.001), with menadione tolerance emphasizes the glutathione-mediated scavenging of ROS (93, 94).

In summary, our culturomic approach using low concentrations of growth substrates or the environmental matrix itself (i.e., water filtrate) in culturing media combined with prolonged cultivation time resulted in the isolation of a broad spectrum of microorganisms. In order to further increase cultivation yields, experimental designs should incorporate strategies that take into account information derived from metagenomic studies, such as those employing genome-informed media, which were already shown to be beneficial for the isolation of symbiotic *Nanoarcheon* species (21) or for the cultivation of Xylella fastidiosa, which requires amino acids for growth (109). Nonetheless, our study contributes to the recent key progress in understanding prokaryotic life, taxonomy, physiology, and ecology which has been made by exploring extreme environments (110), which include Jáchymov’s radioactive springs studied here. Our results confirm that microorganisms living under extreme conditions bear various adaptive or coping mechanisms to thrive under selective pressure and can thus provide a wide spectrum of capabilities potentially exploitable in diverse scientific, biotechnological, or medical disciplines (111). In the past, radiotolerant cultures have been found to be useful in several biotechnological applications, such as bioremediation of nuclear waste, bioethanol production, or for medical purposes such as an acquisition of UVR-protective or desiccation-protective compounds further used in the prevention of skin cancer (112). Therefore, the future microbial exploration of distinct extreme environments should stay in the spotlight, not only as cultivation-independent studies, but also as studies focused on culturomics and cultivation improvements.

## MATERIALS AND METHODS

### Site description and sample collection.

The 4 radioactive water springs Agricola (water resource yield 10 l/min., temperature 29°C, radioactivity 20 kBq/l), Běhounek (300 l/min., 36°C, 10 kBq/l), C1 (30 l/min., 29°C, 11 kBq/l), and Curie (30 l/min., 29°C, 5 kBq/l) spring up at a depth of 500 m below the surface of gneiss mountain bedrock, in the former silver-uranium mine Svornost (Jáchymov, Czech Republic; GPS 50°22′22.9″N 12°54′42.1″E). The chemical composition of the water springs is summarized in Table S1. Under sterile conditions, 20 liters of each spring water was collected and separately filtered with low vacuum through Merck Millipore Sterito Sterile Vacuum Bottle-Top Filters with PES filter membranes with a 0.2 μm pore size (Fisher Scientific). Filtered water (filtrate) was preserved for further experiments overnight at 28°C or 37°C (according to the spring’s temperature). Filter membranes with retained cells were excised from the plastic cup using a sterile pair of tweezers, placed into 25 mL of sterile filtrate of the respective spring water, and incubated for 48 h at 120 rpm.

### Media preparation.

Six nutrient-poor media imitating the original conditions of the water springs and 2 nutrient-rich media were used in this study (Table 1). The nutrient-poor media were prepared as follows: the filtrate of each spring, acquired in the previous filtration step, was supplemented with acetate/lactate/succinate to a final concentration of 0.05%_wt/vol_ or was left unsupplemented with any carbon source, and was solidified with extra pure Noble agar (Difco) at a final concentration of 1.8%_wt/vol_. These media are hereinafter referred to as acetate medium, lactate medium, succinate medium, and non-supplemented medium, respectively. The fifth nutrient-poor medium, an Autotrophic medium containing mineral salts and citrate, was prepared according to Soriano and Walker, 1968 (113). The sixth nutrient-poor medium, a Thiobacillus agar (DSMZ, no. 36m), and 2 nutrient-rich media, 10x diluted Reasoners' 2A medium (1/10 R2A; Merck) and FRR 6, FRR 8 agar (DSMZ no. 822, herein referred to as FRR), were prepared according to the manufacturers' instructions, except that the distilled water was replaced with the filtrate.

### Inoculation and cultivation.

After 48 h, each filtrate with cells released from a filter membrane was used as an inoculum. One hundred μL of the original, 10x, 100x, and 1,000x diluted inoculum were spread evenly over the 8 types of solid media, each dilution in triplicates. Inoculated and sealed plates were incubated at 28°C or 37°C, according to the respective water spring’s temperature, for 6 to 14 weeks until the appearance of colonies.

### Collection and identification of isolates.

Grown colonies were transferred onto fresh 10x diluted Plate count agar medium (1/10 PCA, Merck) and subcultured until pure cultures were obtained. These were then identified by MALDI-TOF MS using a MALDI Biotyper 3.1 Autoflex speed MALDI-TOF mass spectrometer (Bruker Daltonics). The subsequent dereplication was carried out based on the approach described in Strejcek, 2018 (114). Briefly, mass spectra obtained with the MALDI Biotyper were clustered using 0.90 cosine similarity cut-off to mimic bacterial species delineation. A random member was chosen from each cluster as a representative culture. The validity of dereplication was confirmed by subsequent identification of representative cultures based on the 16S rRNA gene sequencing.

Genomic DNA of dereplicated isolates was isolated by thermal lysis (by resuspending an entire microbiological loop of biomass in water for molecular biology and heating it at 98°C for 20 min) or, for hardly lysable cultures, with the aid of a PureLink Genomic DNA Kit (Invitrogen) according to the manufacturer’s instructions. The nearly complete 16S rRNA gene was amplified with 8-27F primer (5′-AGAGTTTGATCMTGGCTCAG-3'') (115) and 1509 −1492R primer (5′-GYTACCTTGTTACGACTT-3′) (115) in 15 μL reactions containing: 7.5 μL KAPA HiFi HotStart Mix 2x (KapaBiosystems, Roche; 0.02U/μL; mixture already contains Mg^2+^ and dNTPs); 0.3 μM primers (Sigma-Aldrich, UK); 1 μL of DNA template (5-20 ng/μL); and 6,41 μL of water for molecular biology (Sigma-Aldrich, UK). The PCR conditions were as follows: 5 min at 95°C; 25 cycles of: 20s at 98°C, 15s at 56°C, 15s at 72°C; 5 min of final extension at 72°C. Amplification was verified on 1% agarose gel. Where there were insufficient DNA yields, this was followed by a second round (reconditioning run) in which the concentration of chemicals was analogical to the first run, differing only in the final volume (25 μL) and in the DNA template (2.5 μL of previous PCR product) (116). PCR conditions were also analogical to those of the first run, only the number of cycles was decreased to 8 to 12 depending on the amplification efficiency in the first run. The obtained amplicons were purified with the aid of a DNA Clean and Concentrator kit (Zymo Research), sequenced unidirectionally with 926-907R (5′-CCGYCAATTYMTTTRAGTTT-3′) primer (117), and subjected to Sanger sequencing. All sequences were trimmed and aligned in MEGAX (118) and subsequently identified against the RefSeq RNA database (NCBI) and EzBioCloud 16S rRNA gene sequence database (119). Where there was <99% sequence similarity to the 16S rRNA gene of the closest type strain, another round of sequencing was performed with 1509R-1492R primer (5′-GYTACCTTGTTACGACTT-3′) to obtain a nearly complete 16S rRNA gene sequence. Isolates were stored as 25% glycerol stocks at −80°C.

In order to assess the evolutionary relationship of dereplicated Jáchymov’s isolates, obtained partial 16S rRNA gene sequences of isolates were aligned using the AlignSeqs and StaggerAlignment functions from the package DECIPHER R (120) (v.2.20.0), while taking into consideration the secondary RNA structure. Since the individual sequences were of different lengths, the resulting multiple sequence alignment (MSA) was manually trimmed to contain only the shared sequence region. The resulting MSA consisted of 83 unique sequences and was 677 positions long, effectively covering the V2–V4 regions of the 16S rRNA gene. The phylogeny reconstruction was done using IQtree (v.2.1.2) ModelFinder chose TIM3e+I+G4 as the best DNA model according to the Bayesian Information Criterion. The phylogenetic tree was visualized and annotated using ggtree. The tidyverse packages were also used for creating accompanying bar plots. All statistical analysis were performed in R.

### Taxonomic classification of potentially novel taxa.

All isolates with ≤98.65% 16S rRNA gene sequence similarity to their closest type strain were subjected to whole-genome analysis to confirm their taxonomic novelty (121). For the isolation of genomic DNA, all strains were grown for 24–72 h on solid 1/10 PCA, R2A, or FRR. Cultures were harvested and the genomic DNA was isolated using a PureLink Genomic DNA Kit (Invitrogen) following the manufacturer's instructions. The high-quality DNA was processed for the whole genome library preparation according to Lopez-Echartea, 2021 (122). The obtained libraries were pooled in equimolar concentrations prior to sequencing in an Oxford Nanopore Technologies MinION platform using a FLO-MIN106 flow cell. Guppy (v3.2.10) was used to perform the base-calling of nanopore sequencing signals, which was followed by Porechop- and Filtlong-based quality filtering (https://github.com/rrwick/Porechop; https://github.com/rrwick/Filtlong), which included removal of the sequencing adaptors, low-quality reads (≤q10), chimeric sequences, and filtering based on the length of the reads (≤500bp). The quality-filtered reads were used for genome assembly using the Canu assembler (v1.7) (123). As the nanopore data are prone to a high sequencing error rate, the genome assemblies need to be polished prior to their downstream analysis. The polishing of the Canu assemblies was carried out using Medaka (v1.4.3) (https://github.com/nanoporetech/medaka). CheckM, available on the Kbase server, was used to assess the quality and the completeness of the genome assemblies (124, 125). Genome annotations for the inference of the functional potential of the bacteria was carried out using the combination of Prokka and PATRIC online server (126–128). The taxonomic characterization of the potentially novel bacterial strains was initiated by extracting the 16S rRNA genes from the obtained genomes. To confirm the validity of the 16S rRNA gene sequences extracted from genomes, a manual comparison to the sequences obtained by Sanger sequencing of the 16S rRNA gene amplicons from individual isolates was performed. Both methods resulted in identical sequences. The 16S rRNA gene sequences were searched against the EzTaxon database (https://www.ezbiocloud.net) (119) to assess the taxonomic affiliations and to retrieve sequences of the validly published closely related strains. The phylogenetic relationship of the strains under study and the associated strains was determined using the UBCG pipeline, which applies the approach of core gene-based phylogeny reconstruction (129). Additionally, the potential taxonomic novelty of the strains was further assessed by analyzing both average nucleotide identity (OrthoANI) and the percentage of conserved proteins (POCP) (130, 131). The genome assemblies generated in this study were submitted to the NCBI Genome Archive and can be accessed under Bio Project ID PRJNA782494 or with the genome accession number as enlisted in Table 2.

### Determination of oxidative stress response.

In order to assess the response of new isolates to oxidative stress, the oxidative stress inducers selection and the inhibition zone assay were performed according to Vatanaviboon, 2002 (36), with subtle adjustments. Briefly, all examined isolates were subcultured in liquid R2A medium (starting OD_600nm_ ~ 0.05) and aerobically cultivated at 28°C or 37°C (according to the isolate’s origin) until the log or late log-phase (OD_600nm_ ~ 0.5, 8–120 h of cultivation, depending on the isolate). After that, 0.5 mL of the log-phase culture was mixed with 4.5 mL of R2A molten agar (0.7%_wt/vol_, ~ 50–55°C) and overlaid on solid R2A medium plates (1.8%_wt/vol_ agar). Menadione 80 mM (2-methyl-1,4-naphthoquinone; Merck) and hydrogen peroxide 9.8 M (30%_wt/wt_; Merck) were used as oxidative stress-inducing agents. Sterile 6 mm paper discs (Merck) were soaked with 5 μL of a stress agent and placed onto completely solidified R2A medium with spread culture. Plates were then cultivated at 28°C or 37°C according to the isolate’s origin. The type strain D. radiodurans DSM 20539^T^, which exhibits a strong resistance to oxidative stress, including that induced by hydrogen peroxide (60), was used as a reference strain to determine the resistance/sensitivity of the isolates to oxidative stress. Growth inhibition zones were evaluated as the inhibition zone radius (mm) after 1–5 days of cultivation.

### Oxidative stress tolerance-associated genes in phylogenetically novel taxa.

The genomes of identified phylogenetically novel taxa were screened for the presence of genes involved in protecting against oxidative damage by various ROS, such as those encoding for catalases, metal-catalases, alkyl hydroperoxide reductases, and organic hydroperoxides as well as carotenoid biosynthesis enzymes, DNA repair enzymes, glutathione, and other regulatory genes, including those encoding the redox-sensitive transcriptional activator SoxR and hydrogen peroxide-inducible genes activator OxyR. These genes collectively account for the EDM in the radiotolerant type strain D. radiodurans (39). Radiotolerant type strain D. radiodurans DSM 20539^T^ was used as a control for the subsequent analyses. The genes involved in oxidative stress tolerance were retrieved from the genome of D. radiodurans DSM 20539^T^, and were searched against the genomes of phylogenetically novel bacterial isolates using blastp. The number of stress response genes among the bacterial isolates was visualized using the software STAMP (132). Spearman's rank correlation was computed to describe the statistical relationship between the response of bacterial isolates to menadione and hydrogen peroxide (i.e., phenotype) and the presence or absence of stress response genes (i.e., genotype). The correlation analysis was preceded by: i) transforming the values (zone of clearance) of the response of bacterial isolates to menadione and hydrogen peroxide to the inverse scale by negating the values, and ii) transforming the stress responses gene copy number to 0 or 1. The data visualization was performed in R using the R packages ggplot2 and corrplot.

### Data availability.

The 16S rRNA gene partial sequences of obtained isolates are available in GenBank under accession numbers OP012574-OP012694. The genome assemblies generated in this study were submitted to the NCBI Genome Archive and can be accessed under Bio Project ID PRJNA782494 or with the genome accession number as enlisted in Table 2.
