# Supplementary material for: Culturomics of Bacteria from Radon-Saturated Water of the World’s Oldest Radium Mine
Source: Microbiol Spectr. 2022 Aug 24;10(5):e01995-22. doi: 10.1128/spectrum.01995-22 (PMC9602452; doi:10.1128/spectrum.01995-22)

Figure S1: UBCG based core-genome phylogenetic analysis (targeting 92 housekeeping genes) of strain *Parviterribacteraceae* sp. J379 with its closest members from the family *Parviterribacteraceae*.

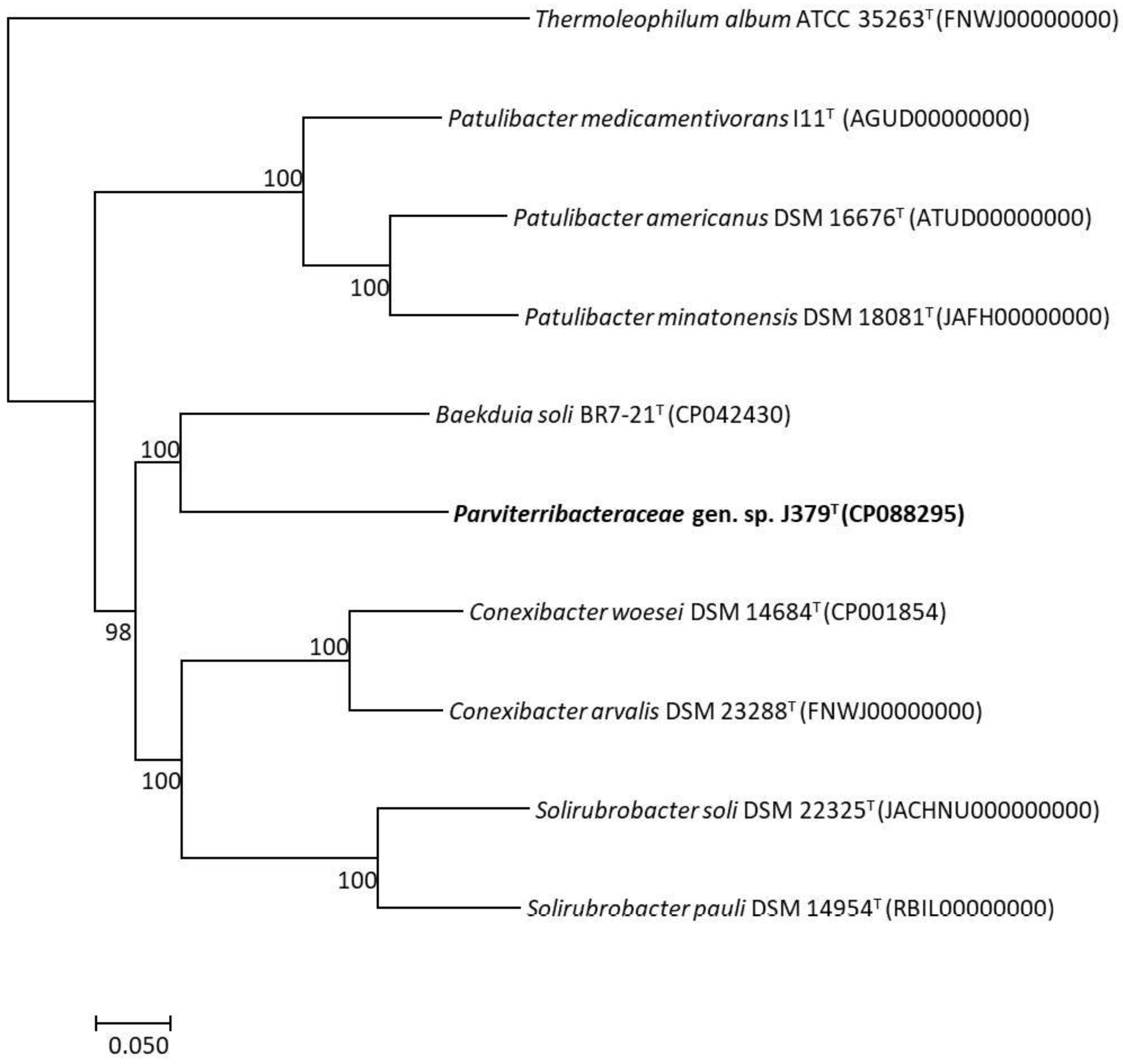

Figure S2: UBCG based core-genome phylogenetic analysis (targeting 92 housekeeping genes) of the strain *Rhizobacter* sp. J219 with its closest members from the family *Comamonadaceae*.

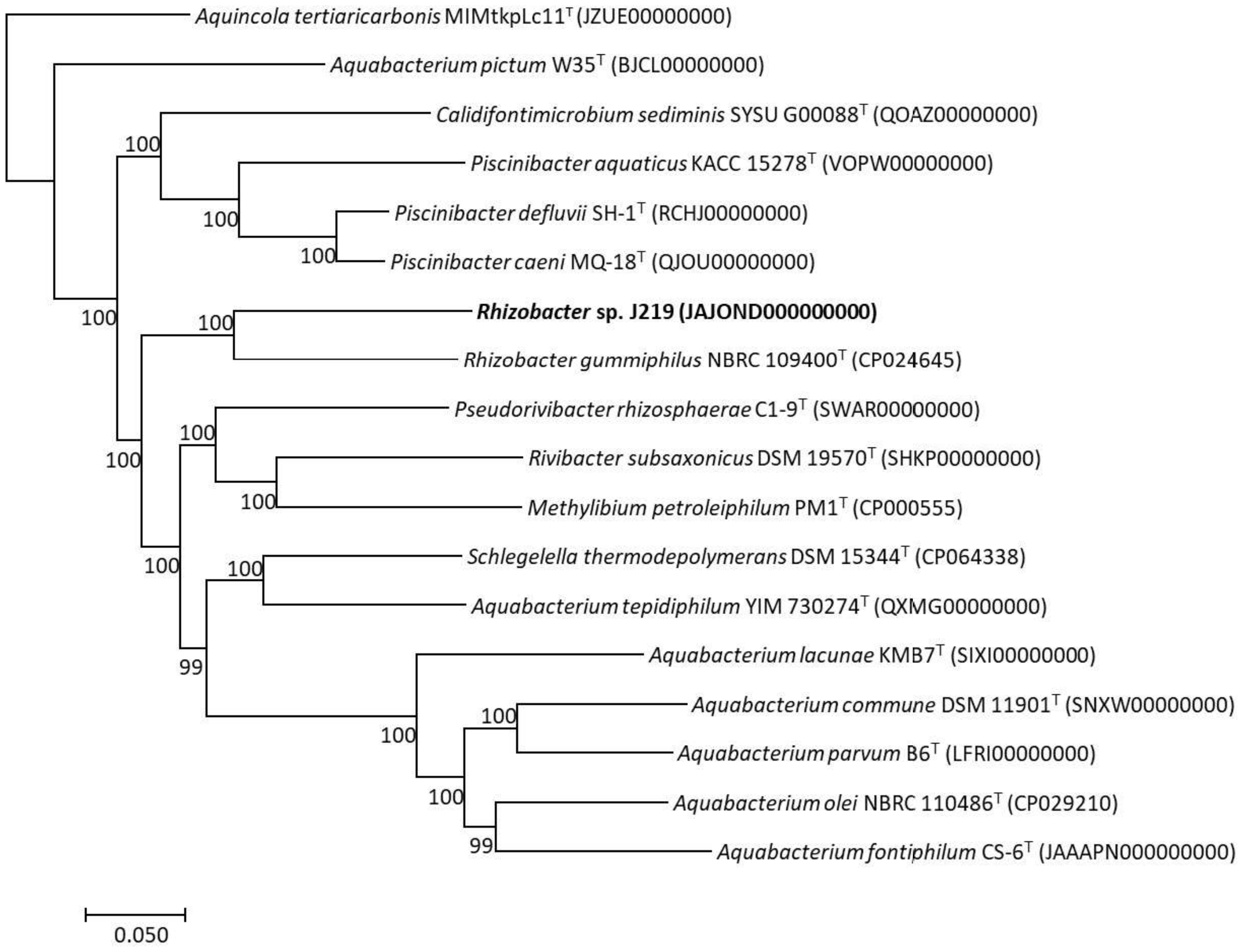

Figure S3: UBCG based core-genome phylogenetic analysis (targeting 92 housekeeping genes) of strain *Aquabacterium* sp. J223 with its closest members from the family *Comamonadaceae*.

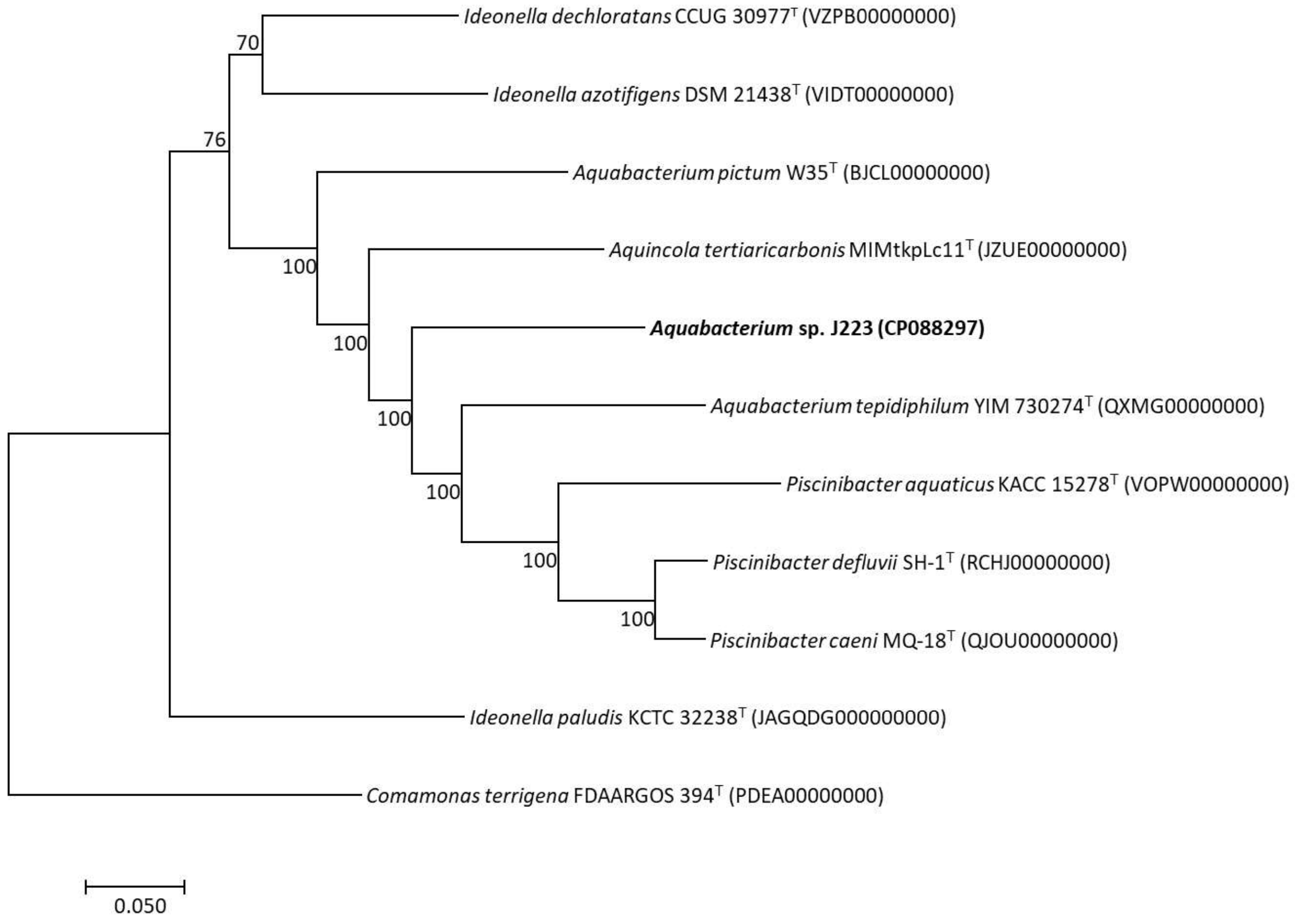

Figure S4: UBCG based core-genome phylogenetic analysis (targeting 92 housekeeping genes) of strain *Aquincola* sp. J276 with its closest members from the family *Comamonadaceae*.

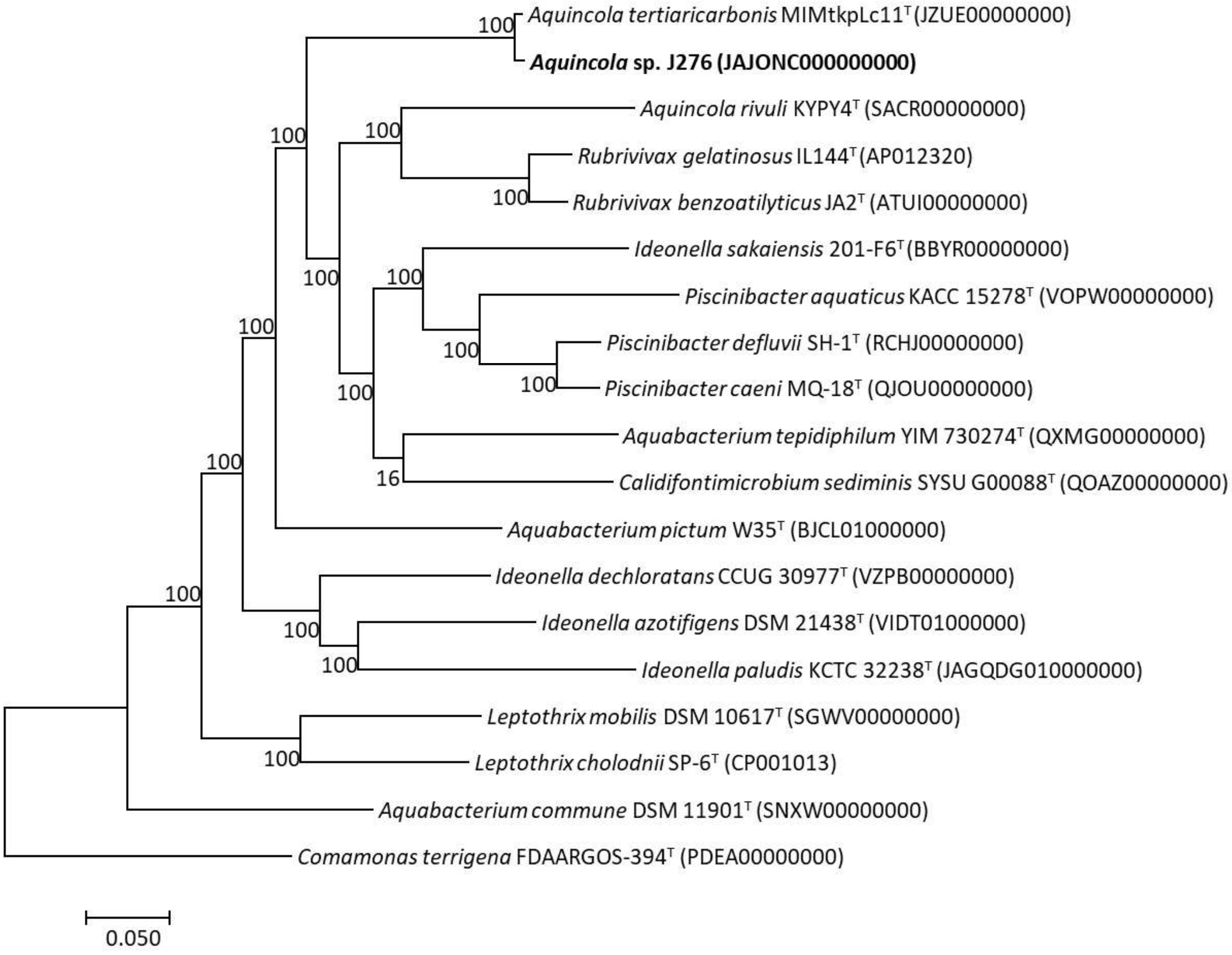

Figure S5: UBCG based core-genome phylogenetic analysis (targeting 92 housekeeping genes) of strains *Sphingomonas* sp. J315 and *Sphingomonas* sp. J344 with its closest members from the family *Sphingomonadaceae*.

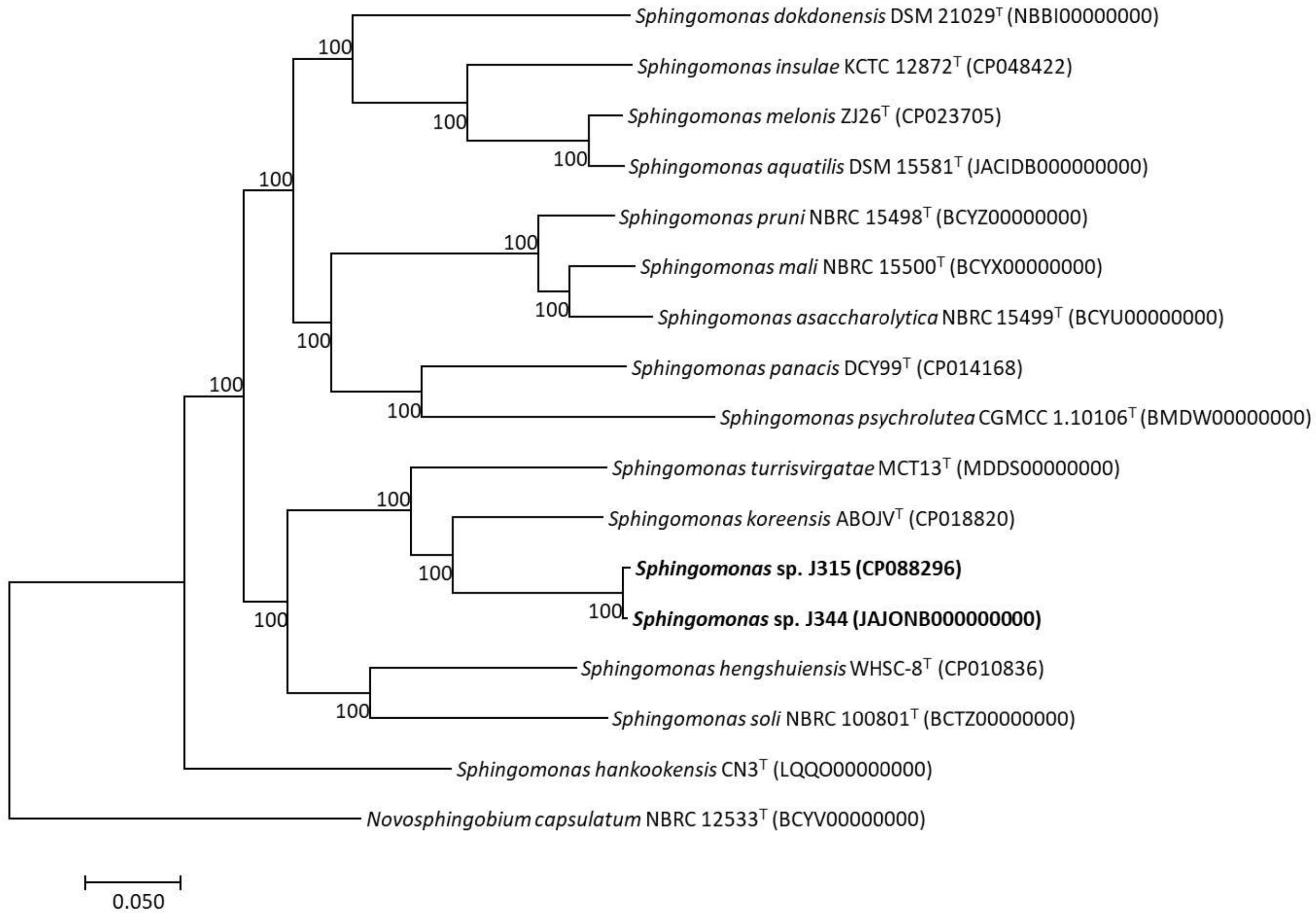

Figure S6: UBCG based core-genome phylogenetic analysis (targeting 92 housekeeping genes) of strain *Phenylobacterium* sp. J367 with its closest members from the genus *Phenylobacterium*.

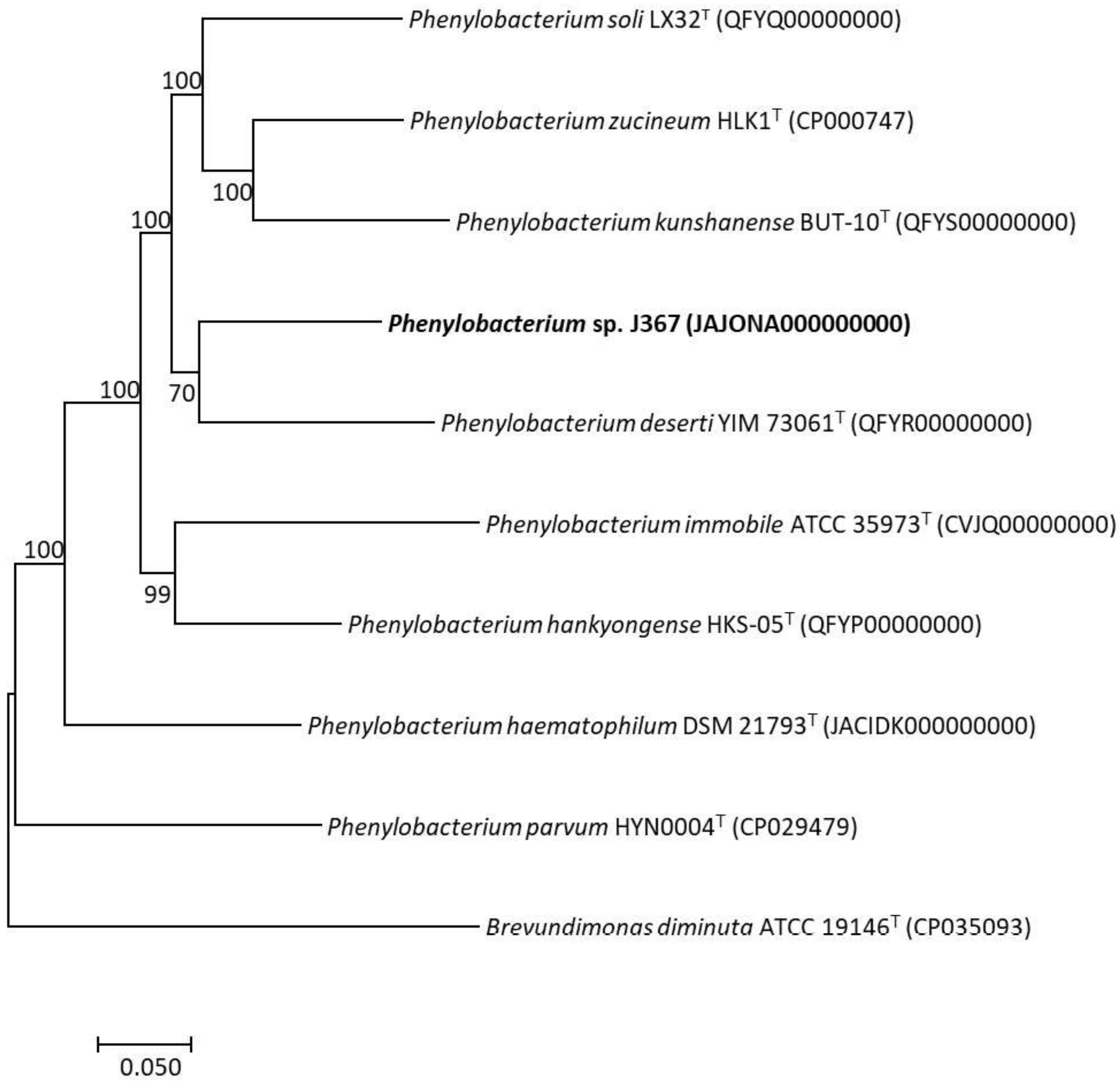

Figure S7: UBCG based core-genome phylogenetic analysis (targeting 92 housekeeping genes) of strain *Phenylobacterium* sp. J426 with its closest members from the genus *Phenylobacterium*.

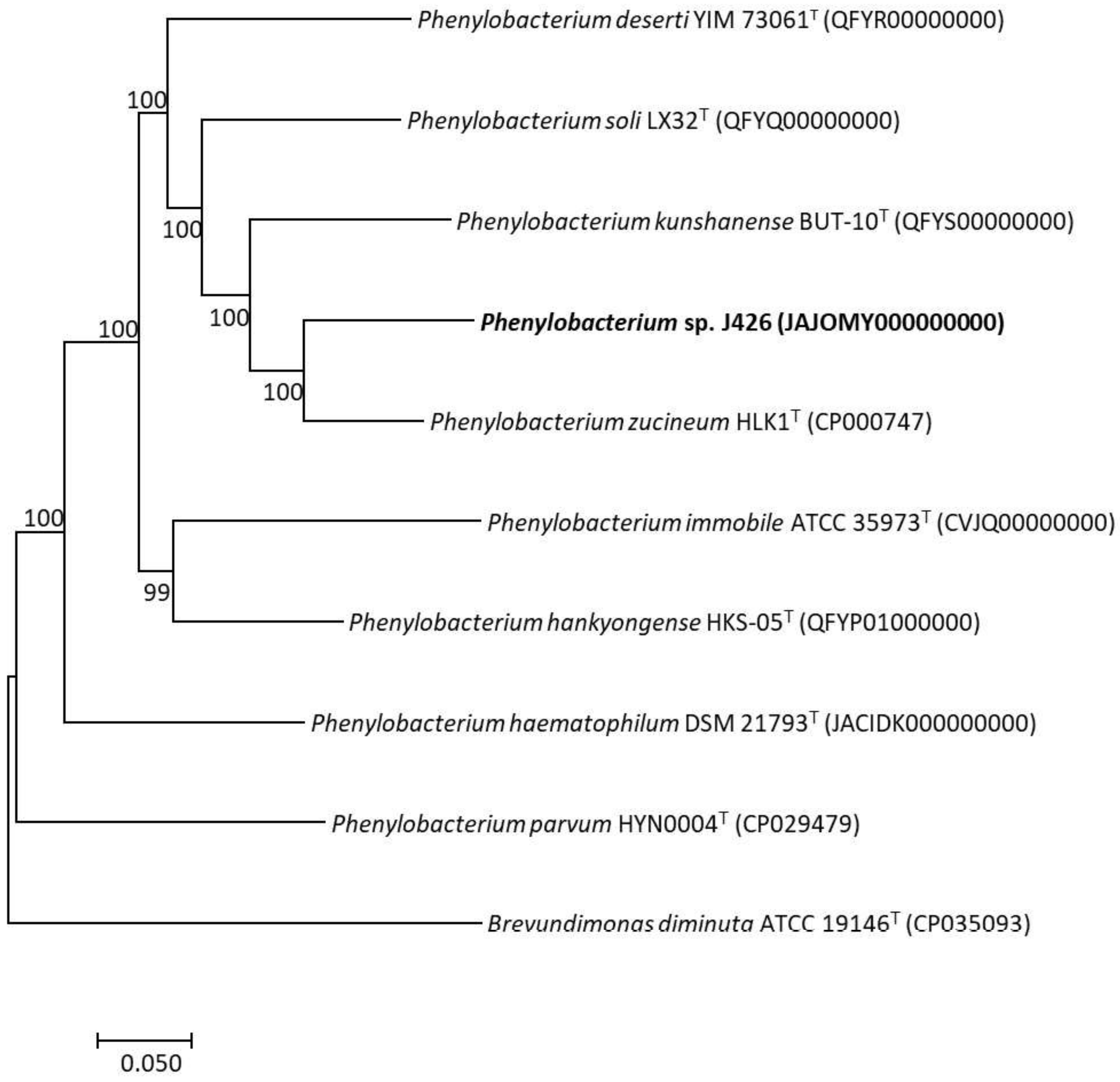

Figure S8: UBCG based core-genome phylogenetic analysis (targeting 92 housekeeping genes) of strain *Mesorhizobium* sp. J428 with its closest members from the genus *Mesorhizobium*.

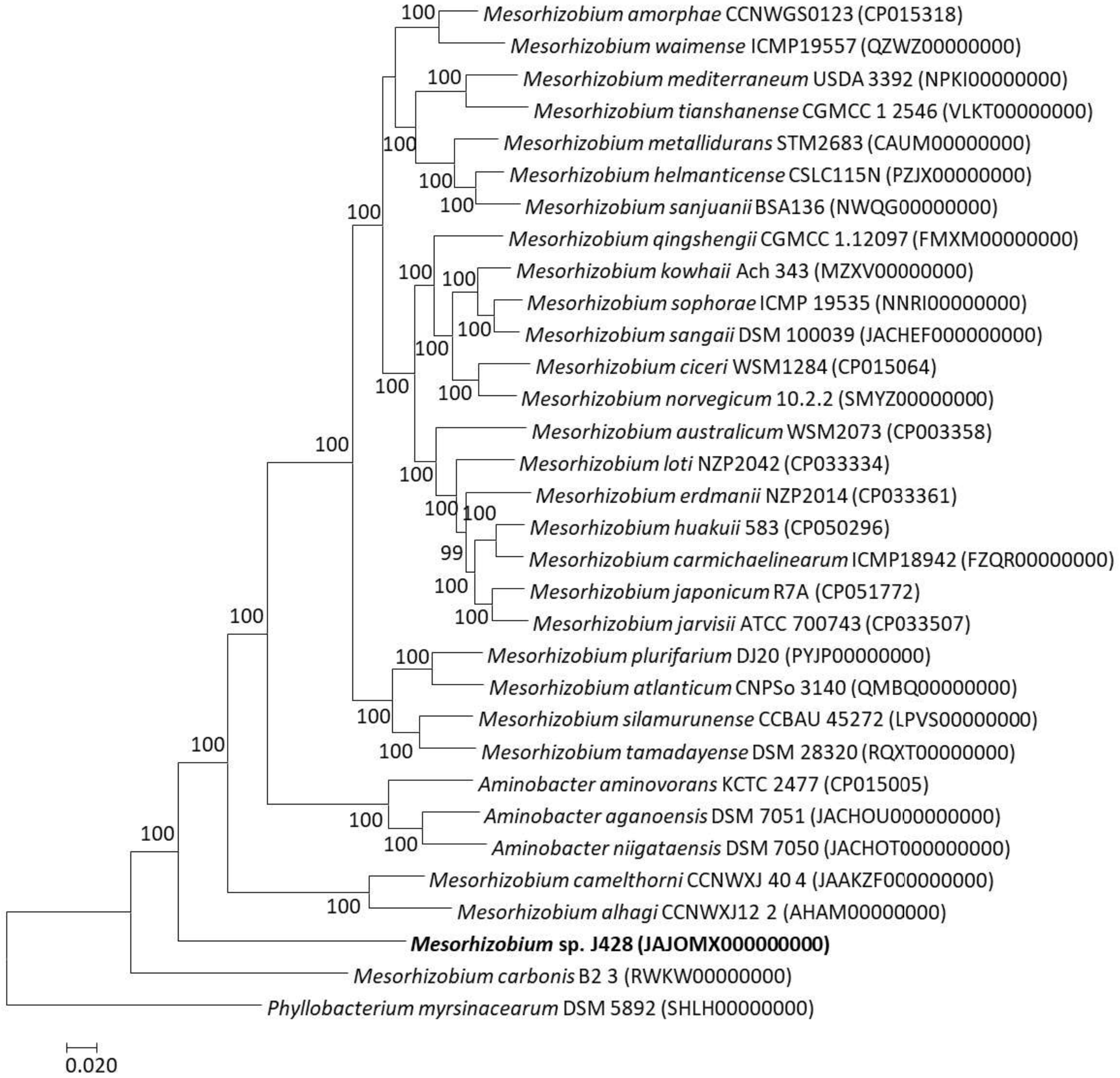

Figure S9: UBCG based core-genome phylogenetic analysis (targeting 92 housekeeping genes) of strain *Pseudomonas* sp. J452 with its closest members from the genus *Pseudomonas*.

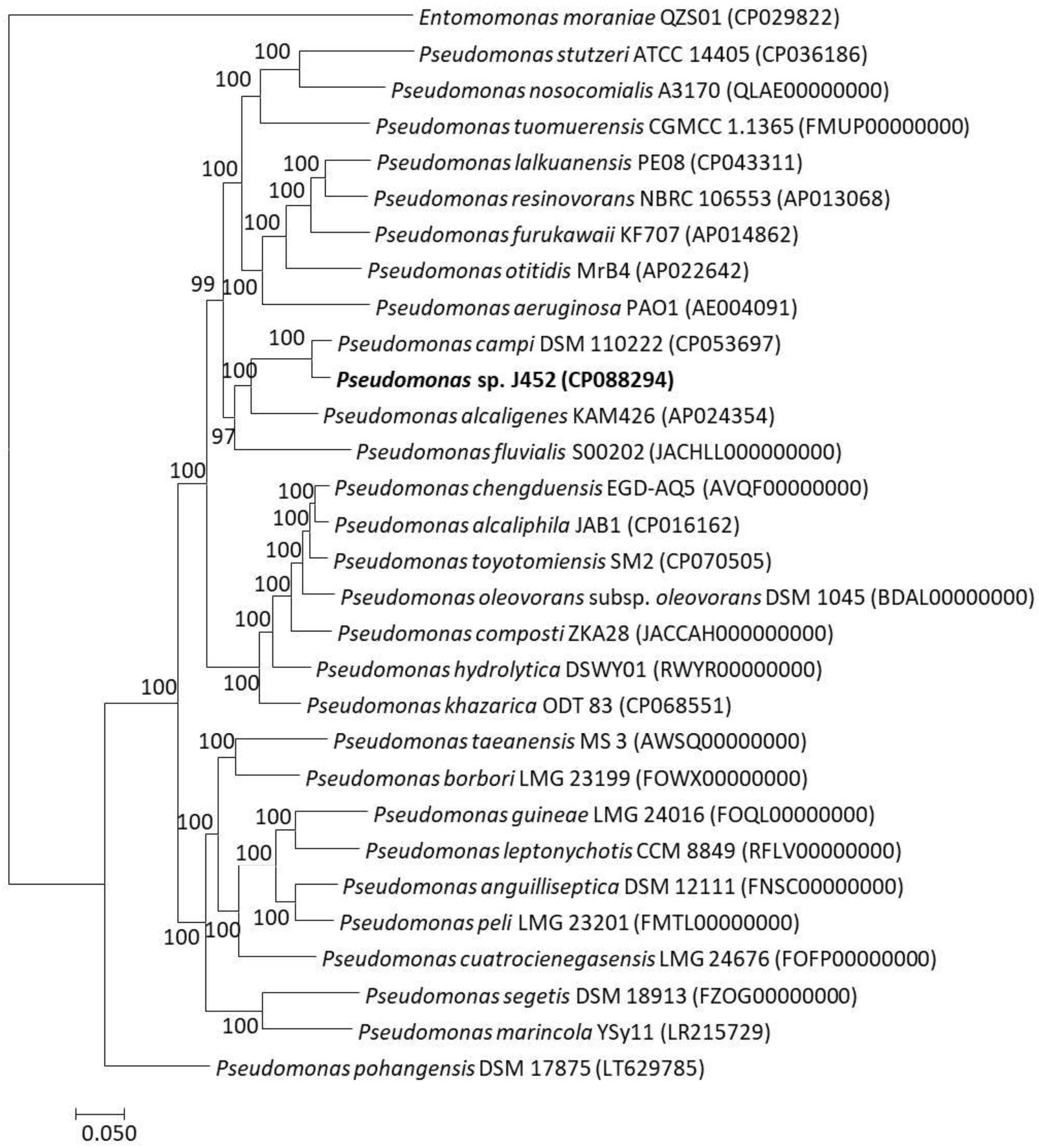

Figure S10: UBCG based core-genome phylogenetic analysis (targeting 92 housekeeping genes) of strain *Hymenobacter* sp. J193 with its closest members from the genus *Hymenobacter*.

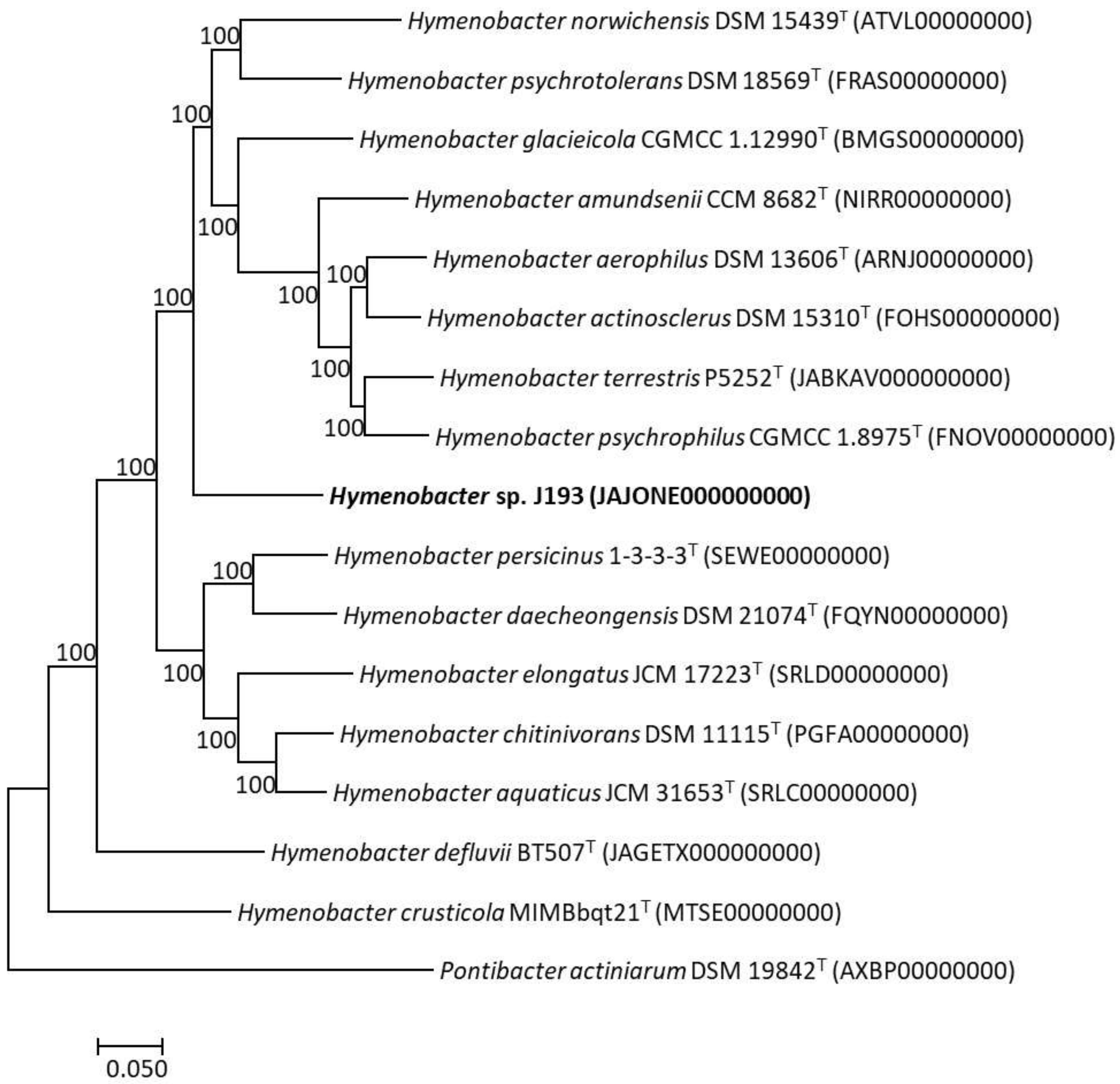

Figure S11: UBCG based core-genome phylogenetic analysis (targeting 92 housekeeping genes) of strain *Flavobacterium* sp. J372 with its closest members from the genus *Flavobacterium*.

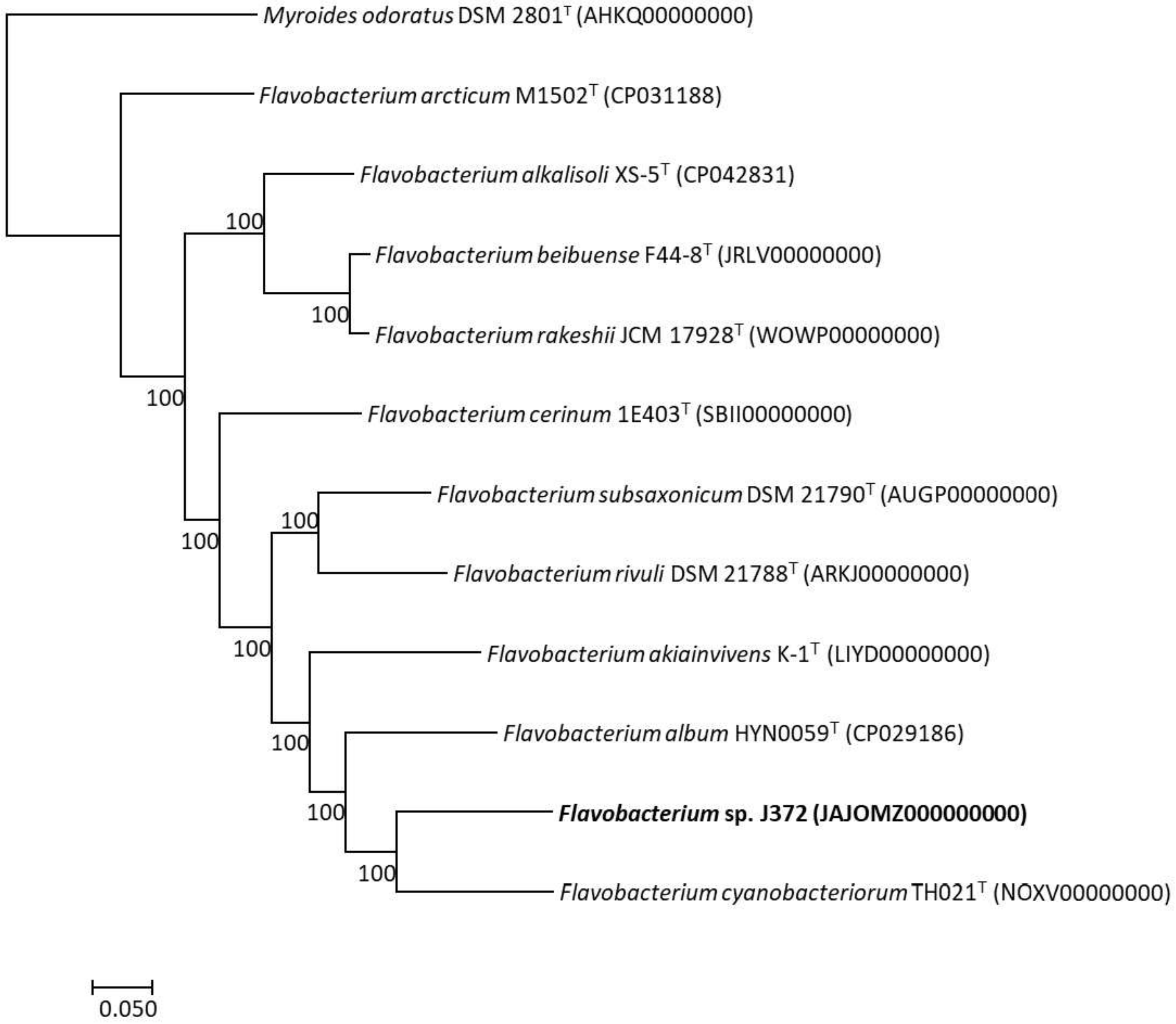

Figure 12: Response to the induced oxidative stress as a zone of clearance upon exposure of the strains to hydrogen peroxide and menadione (a) and prevalence of genes (shown as a gene copy per genome) involved in endogenous defence mechanism (EDM) like synthesis of glutathione (b) and DNA repair (c) adopted by putative novel taxa from the Jáchymov's radon water spring.

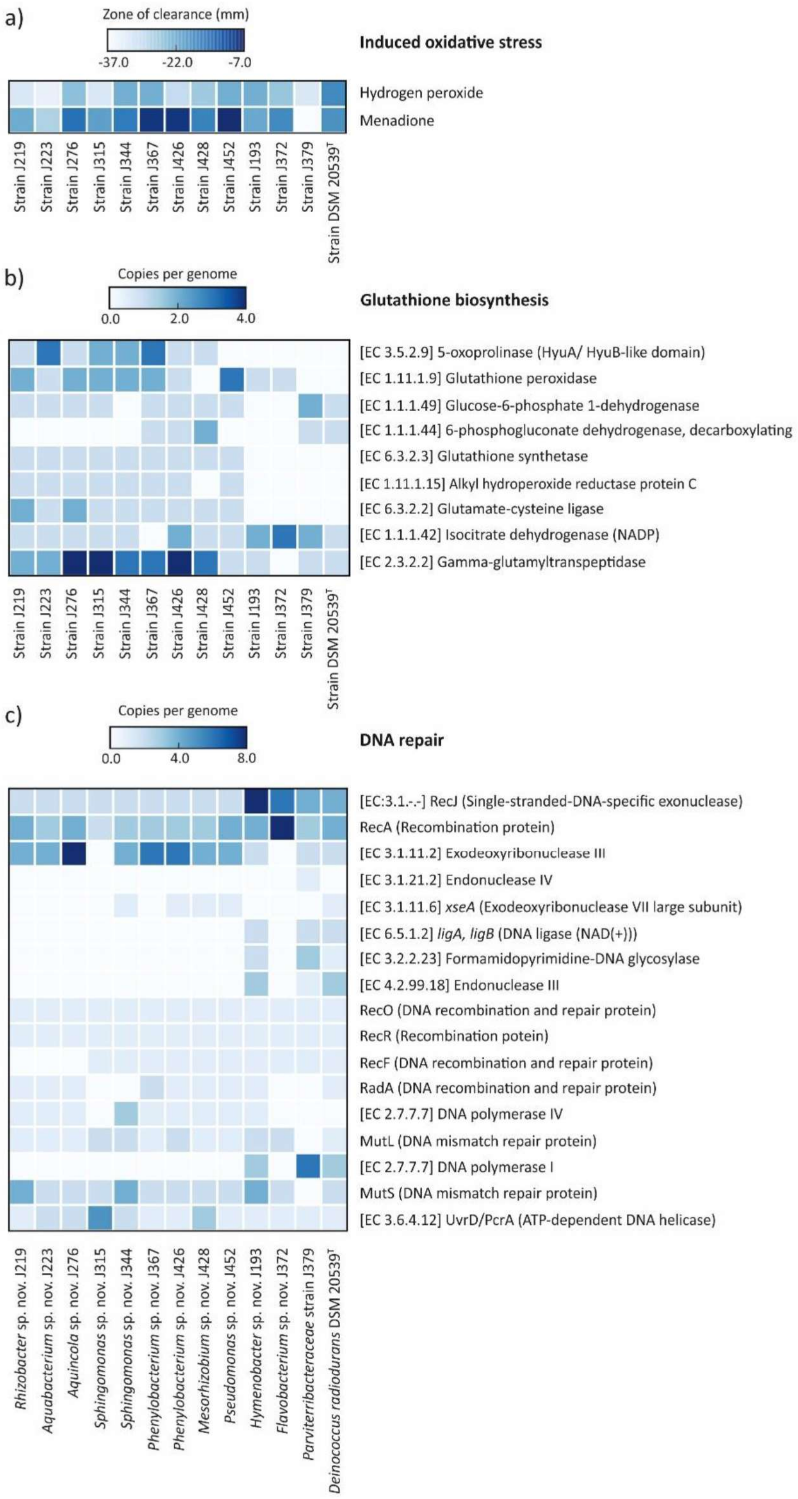

Supplement: Supplemental file 1 — Supplemental figures. Download spectrum.01995-22-s0001.pdf, PDF file, 1.4 MB [file spectrum.01995-22-s0001.pdf]
